# A meta-ethnography investigating relational influences on mental health and cancer-related health care interventions for racially minoritised people in the UK

**DOI:** 10.1371/journal.pone.0284878

**Published:** 2023-05-10

**Authors:** Damien Ridge, Karen Pilkington, Sheila Donovan, Elisavet Moschopoulou, Dipesh Gopal, Kamaldeep Bhui, Trudie Chalder, Imran Khan, Ania Korszun, Stephanie Taylor, on behalf of the SURECAN Investigators

**Affiliations:** 1 School of Social Sciences, University of Westminster, London, United Kingdom; 2 School of Health and Care Professions, University of Portsmouth, Portsmouth, United Kingdom; 3 Barts and The London School of Medicine and Dentistry, Queen Mary University of London, London, United Kingdom; 4 Department of Psychiatry, Nuffield Department of Primary Care Health Sciences, University of Oxford, Oxford, United Kingdom; 5 Wadham College, University of Oxford, Oxford, United Kingdom; 6 World Psychiatric Association Collaborating Centre, Oxford, United Kingdom; 7 Department of Psychological Medicine, Kings College London, London, United Kingdom; University of Alabama at Birmingham, UNITED STATES

## Abstract

**Objective:**

Despite calls to increase the ‘cultural competence’ of health care providers, racially minoritised people continue to experience a range of problems when it comes to health care, including discrimination. While relevant qualitative meta-syntheses have suggested better ways forward for health care for racialised minorities, many have lacked conceptual depth, and none have specifically investigated the relational dimensions involved in care. We set out to investigate the social and cultural influences on health care interventions, focusing on psychological approaches and/or cancer care to inform the trial of a new psychological therapy for those living with or beyond cancer.

**Method:**

A meta-ethnography approach was used to examine the relevant qualitative studies, following Noblit and Hare, and guided by patient involvement throughout. Papers were analysed between September 2018 and February 2023, with some interruptions caused by the Covid pandemic. The following databases were searched: Ovid MEDLINE, EBSCO CINAHL, Ovid Embase, EBSCO PsycINFO, Proquest Sociology Collection (including Applied Social Sciences Index & Abstracts (ASSIA), Sociological Abstracts and Sociology Database), EBSCO SocINDEX, Ovid AMED, and Web of Science. The systematic review protocol was registered with the International Prospective Register of Systematic Reviews (PROSPERO) (ID: CRD42018107695), and reporting follows the eMERGe Reporting Guidance for meta-ethnographies (France et al. 2019).

**Results:**

Twenty-nine journal papers were included in the final review. Themes (third-order constructs) developed in the paper include the centrality of the patient-practitioner relationship; how participants give meaning to their illness in connection to others; how families (rather than individuals) may make health decisions; how links with a higher power and spiritual/religious others can play a role in coping; and the ways in which a hierarchy of help-seeking develops, frequently with the first port of call being the resources of oneself. Participants in studies had a need to avoid being ‘othered’ in their care, valuing practitioners that connected with them, and who were able to recognise them as whole and complex (sometimes described in relational languages like ‘love’). Complex family-based health decision-making and/or the importance of relations with non-human interactants (e.g. God, spiritual beings) were frequently uncovered, not to mention the profoundly emergent nature of stigma, whereby families could be relatively safe havens for containing and dealing with health challenges. A conceptual framework of ‘animated via (frequently hidden) affective relationality’ emerged in the final synthesis, bringing all themes together, and drawing attention to the emergent nature of the salient issues facing minoritised patients in health care interactions.

**Conclusion:**

Our analysis is important because it sheds light on the hitherto buried relational forces animating and producing the specific issues facing racially minoritised patients, which study participants thought were largely overlooked, but to which professionals can readily relate (given the universal nature of human relations). Thus, training around the affective relationality of consultations could be a fruitful avenue to explore to improve care of diverse patients.

## Introduction

The problems that racially minoritised people (i.e. people “actively minoritised by others rather than naturally existing as a minority” [1] (p. e419)) associate with health care—like stigma, discrimination and unempathic treatment—are well documented [2, 3]. However, professional health care training appears not to readily assuage these problems. One issue with Western health care (that privileges white claims to knowledge) is that racially minoritised people are readily positioned as ‘other’ [4]. This approach can decide what the problems are for (rather than in consultation with) racially minoritised people, sidelining the hidden disadvantages people face. For example, one study identified that the mere seeking of health care by South Asian minorities could put at risk their safety and cultural identities [5]. In response, some researchers have called for deeper ways of thinking through the issues involved. For example, a kind of ethnographic approach to clinical care, whereby practitioners are encouraged to enter into the worlds of racially minoritised people with the curiosity of anthropologists, was suggested by one group of scholars [2]. Nevertheless, it is clear that better ways of conceptualising racially minoritised health care are needed. We began this meta-ethnography with an interest in the social and cultural influences on cancer-related psychological interventions to inform our work on developing and evaluating an intervention for people living with and beyond cancer experiencing psychological distress [6]. The process of conducting this meta-ethnographic review led to the elaboration of the concept of ‘animated by (frequently hidden) affective relationality’ in the final synthesis, tying all the themes together. We briefly introduce ‘affective relationality’ below as a prelude.

### Affective relationality

The current “relational turn” among wide ranging disciplines (e.g. psychiatry, sociology, archaeology, philosophy and critical psychology) has roots in older symbolic interactionist and psychoanalytic thinking [7, 8]. What does it entail to think in epistemologically ‘relational’ terms? Moving past old arguments of individualism versus collectivism, we focus instead on relational categories, i.e. selves as emergent via social interactions, as ‘interactants’ rather than individuals, where personal agency relies on the configurations of our relationships [7, 9]. Additionally, the role of non-human things (e.g. the NHS) are important considerations in the way that relations develop and are meaningful [10]. There is an ‘affective’ dimension to relationality [11], which adds drama and intensity. We are all vulnerable, whether patients or health professionals [12]. We are drawn into and influenced by the emotional experiences evoked by our encounters with others, where the stakes are high because, *“…we are [in] constant danger of our self being challenged*… *[others] not providing the support we expect; or using our relationships to harm us*.*”* (p. 29) [13]. Thus, in relational thinking, individual agency is emergent and possible only via our interdependencies, and not something that individuals can possess on their own [14, 15]. Another way of saying it, is that relations can be considered to *constitute* our lived realities, they are not just an interesting aspect of social life to study [10].

### Previous meta-syntheses

There have been various qualitative meta-syntheses of relevance to the focus in our paper on improving mental health and cancer-related health care interventions for racially minoritised people. While some syntheses tended to treat different minority groups as a single cultural minority category [16, 17], there were notable exceptions, whereby diverse groups were identified separately (or as much as possible, as original studies do not always make such distinctions) [18, 19]. Some relevant syntheses appeared to highlight a deficit model at times, whereby minority groups were cast as somehow ‘other’ e.g. belief systems considered ‘superstitions’ [18]. Other syntheses focused more on experiences outside of health care, or issues about accessing care, rather than on our focus on achieving a deeper understanding of care experience itself [18, 20, 21].

One synthesis of studies involving South Asian service users did touch upon interactional issues (e.g. families as decision-makers) [5]. Another study considering relationships was pitched at the descriptive level, and thus did not offer a conceptual framework of those relations [16]. Other syntheses focused on care experiences (e.g. lack of support for distress), but not broadly, e.g. by focusing on men with prostate cancer [3, 19]. Conceptual insights of relevance to interactivity, are however, offered up in this literature. For example one study developed the concept of ‘recursivity’, the back and forth interaction between people and providers involved in candidacy (i.e. how people’s sense of being eligible for care is constructed via their health interactions), as a way of highlighting how interactions shape the possibilities of health care [21]. While moving in a relational direction, this study did not specifically develop relationality conceptually. In sum, while some syntheses advocated improving professional ‘cultural competency’, and even put forward conceptual models [16, 17], or were especially rich in conceptual synthesis [21], they only reference relationality in passing.

Our meta-synthesis informed the SURECAN research trial of a psychological intervention for those living with and beyond cancer. SURECAN develped a psychological intervention based on Acceptance and Commitment Therapy to improve quality of life (QoL), with added options for exercise and work support (ACT+) [6]. Previous qualitative meta-syntheses of patients’ views about life after cancer and treatments to improve QoL focused on survivorship, exercise and return to work [22–24]. Any understanding of issues that might arise when racially minoritised patients, living with and beyond cancer, engage with psychological interventions designed to improve QoL was limited [25]. Thus, the aim of our meta-synthesis was to synthesise experiences of health care/interventions for those living with or beyond cancer, including but not limited to those with a psychological dimension, to inform the design of a UK/NHS based “culturally sensitive” trial intervention that would better appeal across a range of diverse populations (the trial settings included diverse areas like East London). This is a crucial point for talking therapy interventions, like ACT+, in the NHS.

While demand for therapy interventions is increasing among ethnic minorities, there is less evidence supporting their use among different ethnic groups [26]. Cancer care itself was integral to the trial, with any subsequent integration of ACT+ into the NHS being connected to cancer care. Clearly, the perception amongst those using both cancer services and/or mental health services, is of racism and failures to consider cultural and religious diversity [27, 28]. The coverage of both areas (cancer and mental health) in this review was part of the innovative nature of the review, and for which considerable discussion was generated in the research team. For example, many of the psychological therapists employed on the trial were unfamiliar with issues that patients with cancer faced. Our review thus attempted to identify areas of relevance to patients on the trial, eliciting the core themes and issues across psychological and cancer services/interventions. We, therefore, focus on reviewing qualitative studies on care interventions that had a psychological element, and/or were of relevance to those living with or beyond cancer. Our synthesis aimed to answer the research question: What are the important cultural and social influences on mental health and/or cancer-relevant care interventions for racially minoritised people in the UK?

## Methods

The meta-ethnography approach was informed by Noblit and Hare’s [29] seven-step meta-ethnography. The seven stages were: “1. Getting started, 2. Deciding what is relevant to the initial interest, 3. Reading the studies, 4. Determining how the studies are related, 5. Translating the studies into one another, 6. Synthesising translations, and 7. Expressing the synthesis.” The systematic review protocol was registered with the International Prospective Register of Systematic Reviews (PROSPERO) (ID: CRD42018107695) [30], and reporting follows the eMERGe Reporting Guidance for meta-ethnographies [31].

### Search strategy and selection of relevant studies

The search strategy was designed to include terms reflecting culture and ethnicity combined with those reflecting a psychological, mental health and/or cancer-related focus. Searches were conducted of the following databases using a combination of free text and index (MeSH or equivalent) terms: Ovid MEDLINE, EBSCO CINAHL, Ovid Embase, EBSCO PsycINFO, Proquest Sociology Collection (including Applied Social Sciences Index & Abstracts (ASSIA), Sociological Abstracts and Sociology Database), EBSCO SocINDEX, Ovid AMED, and Web of Science. Searching was carried out in September 2018 by an experienced information scientist (KP) from inception to August 2018 using the ‘comprehensive’ approach espoused by France and colleagues [32], to identify all available studies (see S1 Appendix for full search strategy for OVID MEDLINE and other databases). We also carried out iterative searches, checked the references of included studies and asked experts in the field for further relevant studies.

We sought studies of people aged 16 years or over to fit with eligibility for our larger trial. We included all qualitative and mixed-methods studies where the qualitative component was reported in detail. Studies were selected that reported on Black and Minority Ethnic (BME) or Black, Asian and Minority Ethnic (BAME) prevalent in the UK, as well as specific ethnic groups in the UK (according to Office for National Statistics, ONS). We excluded studies where racially minoritised groups were included only as part of a qualitative sampling strategy. Large-scale surveys that include some qualitative data through open questions, as well as analysis of online discussion group content, were also excluded. In effect, semi-structured interviews (one paper only referred to ‘interviews’ but was likely to have been semi-structured in nature [33]), in-depth interviews, and focus groups covered the range of data collection approaches. Sampling was mainly purposive or convenience (although some approaches were not reported), while analyses were mainly thematic in nature, but also included Interpretative phenomenological analysis (IPA), framework analysis and constant comparison. Acknowledging that culture and ethnicity overlap [34], we also looked for research with other disadvantaged or underserved groups in the UK. One paper on survivorship after breast cancer treatment included women from diverse social backgrounds [35], although most women in the study were from racially minoritised backgrounds. In terms of interventions, any form of psychological intervention and/or cancer service was eligible, including cancer/survivorship self-management or self-help, as long as these self-help modalities were part of an intervention.

The search results were imported into EndNote for de-duplication then imported into *Abstrackr* for screening purposes. Two reviewers (DG and KP) independently screened the titles and abstracts of the records to identify studies which appeared to meet the inclusion criteria. Given the very large number of potentially relevant studies located, as well as our overarching aim to inform a service design for cancer-related psychological intervention for use in a contemporary UK trial, we decided i)., to focus on recent studies published after 2010, as well as studies undertaken only in the UK, and ii)., to not investigate studies in languages other than English (or the potential for evidence to be lost due to their exclusion). These were the only substantive differences from the original PROSPERO record. After the initial screening, all potentially relevant studies were obtained as full-texts and assessed for relevance by two reviewers (SD and EM) working independently. Assessments were compared, and disagreements were settled by discussion with a third reviewer (KP) and where necessary, at least one other member of the review team arbitrated (DR). A record was kept of the screening process, the numbers included and excluded, and the reasons for inclusion/exclusion. In terms of papers that appeared to fit the inclusion criteria (for at least one reviewer) but ultimately did not, four such papers were identified and excluded following adjudication by KP and DR. A paper looking at barriers to psychological therapies for Somali, Urdu, Bengali and Tamil groups was excluded because of a lack of focus on interventions and services [36]. One paper investigating barriers to health care presentations with symptoms of cancer in low socioeconomic groups largely consisted of a sample who had never had cancer, and so was excluded [37]. A study of Polish migrants’ decision-making about help-seeking for distress was excluded because experiences of services/interventions was not the main focus, and furthermore, none of the sample had experience of mental health services in the UK [38]. Finally, a study of African Caribbean female experiences of distress was excluded because it too did not focus on experiences of interventions or services [39].

### Data extraction and quality assessment

Two reviewers (SD & EM) independently extracted basic study characteristics using a template that was developed and piloted specifically for this project. The template was adapted from one developed for a previous meta-ethnography [40], with further elements added in consultation with the wider team. A list of the data items extracted is provided in S2 Appendix. After completing the data extraction, data were compared, any discrepancies discussed and a final, corrected version produced. The quality of studies were assessed by two researchers (SD and EM) using the JBI Critical Appraisal Checklist for Qualitative Research [41]. This assessment looked at robustness and validity, and the fields covered the research design, theoretical perspectives, reflexivity and ethics. We did not exclude studies due to quality, however, relative weaknesses/strengths of studies informed the emergent interpretations. Studies scoring at least 7 on the JBI scale are indicated in S1 Table.

### Data synthesis

Meta-ethnography was chosen for the approach to synthesis due to its the focus on developing original conceptual insights and bespoke reporting guidance [42]. The fidelity with primary studies was also considered important by the research team. We considered carrying out a subgrouping of papers, as the number of studies identified was relatively large for meta-ethnography. However, we determined that the synthesis should provide a broad understanding of cultural and social influences on cancer/psychological interventions, focused around lived experience (to which meta-ethnography is well suited, given the prioritisation of participant quotes as outlined below).

The included studies were read and re-read by three reviewers (SD, EM and KP) to identify the recurring concepts related to racially minoritised individuals’ experiences of psychological interventions, cancer services or cancer-related health encounters. For the purpose of this meta-ethnography, we defined recurring concepts as categories of meaning that were evident across multiple papers. Three reviewers (SD, EM, KP) independently identified recurring concepts from participant quotes (first-order constructs) and the related author interpretations (second-order constructs). For each primary study, we extracted data from across all sections of the paper that related to patients as participants (i.e. we did not extract data that related to other study participants such as health professionals or carers).

#### Determining how studies were related

We compared various aspects of the included studies: the focus of the study; type of health service accessed; the severity of the health condition; ethnicity and gender of participants; the findings; key themes; and conclusions. We used an Excel spreadsheet to compare basic study characteristics and participant groups. We explored the relationships between the key concepts arising from different studies using Excel tables that each of the three reviewers independently created. The tables included key concepts from each study with the corresponding participant quotes and author interpretations and explanations to aid comparisons. The studies were represented in columns and the concepts were in rows, thus helping to elucidate the relationship between studies. The data that we extracted and presented under each of the themes were used as the basis for the translation across studies.

As well as comparing the key concepts across the whole data set (of included studies), we also examined how the recurring concepts featured within and across various sub-groups of studies, for example the ‘mental health’ and ‘cancer’ studies, and the ‘severe mental illness’ versus ‘mild/moderate mental illness’ studies to see if these concepts were consistent across different groups rather than being specific to mental health-related experiences for example. We also examined whether the recurring concepts were reflected in papers involving participants of different genders, ethnic groups, and across different time periods and from differing data collection methods. We used the Excel spreadsheet to explore the influence of the different conditions (cancer and mental health), different ethnic groups, and different levels of severity of mental illness. Having identified a recurring concept, we then looked for any disconfirming cases.

#### Translating the studies into one another

We began the translation with 3 key studies which had been identified early in the search process as offering rich data, and which had a primary focus of relevance to our research question but in different patient groups. We compared these and then extracted the concepts from one study and entered these into the Excel spreadsheet with one main concept per line. Gradually, we built a table of similar concepts across these 3 studies before moving onto other studies. Recurring concepts became apparent as the process progressed. We preserved the meanings and contexts of relations between concepts across (and also within) studies by only extracting the authors’ interpretations where these were supported by participant data (quotes) and vice versa. We also extracted the themes and conclusions from each paper and compared these with our extracted data to ensure that we reflected the authors’ perspectives on how the concepts in their study were related. Where possible, our themes and concepts were described using the metaphors and language of the original authors.

In a series of analytical sessions involving the core team (led by DR), the recurring concepts established from the primary studies were presented, discussed and debated. The three reviewers had independently extracted concepts, which meant that variations in how concepts were named and understood were debated until we reached consensus on which concepts recurred across the studies and how each should be labelled. The recurring concepts were grouped into themes. A preliminary analysis, comprising ten key themes was presented to the wider project team for discussion. At this time, work continued on identifying relevant studies, and eight more were subsequently included. The core team undertook further analysis and refinement of the themes before presenting the final analysis, comprising six overarching themes to the wider team (as reflected by the headings in the results section below). In the ensuing discussion, the team linked some of the findings to existing literature and identified some potential conceptual frameworks and interpretations that would explain the findings and reflect third-order constructs (our interpretations of participant quotes and authors’ original interpretations).

We then re-examined the papers in light of our final set of recurring concepts and overarching themes to identify any disconfirming cases. Where these were apparent, we carried out a refutational analysis by comparing the study methods and participants with the aim of finding explanations for these. Importantly, for any disconfirming cases identified that could not readily be explained, these were reflected in the explanation of each theme. We also developed and discussed various visual models as a way of considering the relationship between the themes, as well as moving towards the final synthesis of key/recurring concepts.

#### Synthesising translations and expressing the synthesis

The team used a number of techniques and steps to derive the final synthesis drawing on recommendations from the eMERGe guidance [31], and the experience of other research teams undertaking similar projects [43–45]. The first stage involved the core team of researchers who had been involved in identifying the recurring concepts discussing their understanding of each recurring concept to agree its scope, where there was overlap and how each concept linked to other concepts to generate the overarching themes. The team then presented the agreed concepts and proposed themes to the wider research team for discussion and clarification. The concepts and overarching themes were also presented at several research conferences to audiences including the Society for Academic Primary Care Annual Scientific Meeting (Exeter, 2019) and the Third Victorian Cancer Survivorship Conference (Melbourne, 2020). All feedback from these presentations including questions were noted and circulated to the research team. To develop a synthesis with clear lines of argument, team members (SD, EM, KP and DR) each attempted to produce a visual representations showing the relationships between the themes. Each person presented their diagrammatic representation to the wider research team and similarities across the visualisations were noted while differences were explored. Along the way, various concepts were discussed in an attempt to synthesise all the findings. For example, “social worlds” was an early contender, a concept having a long gestation in the sociological literature. Social worlds draws attention to the way that social interactions themselves—with loosely linked groupings and shared languages—are important in understanding social phenomena, including health [46, 47]. Candidacy theory was also considered (see above), which dynamically focuses on how patient eligibility for health interventions is negotiated between patients and the NHS [25]. While both concepts potentially draw attention to the interactive and co-constructed nature of health consultations, and helped to explain vulnerabilities, they were not able to connect-up and breathe life into all of our findings. Instead we discovered that we needed a fully relational concept. It was through this prolonged and iterative process over several years that it became clear that a framework that emphasised the hitherto (frequently hidden) affective and entangled nature of human experiences, was needed to synthesise our findings in a coherent way.

### Patient and Public Involvement and Engagement (PPIE)

Patient and public involvement and engagement (PPIE) in systematic reviews can increase the usefulness of syntheses of published research [48]. PPIE has been usefully incorporated into qualitative synthesis research previously [49]. Those who have attempted to incorporate PPIE into meta-ethnographies have reported increased insights [40]. Thus, in the current study, emergent findings from the meta-ethnography were initially presented to a group of patients assembled by the Deep End Yorkshire & Humber GP Network, including socio-economically deprived populations, in December 2018. Three of 7 patients in the group were not born in the UK. Each theme identified in the preliminary analysis was presented, along with a short explanation, with patients invited to discuss what each theme evoked. Key patient messages were recorded on post-it notes by the group facilitator, who asked participants to prioritise messages to inform our paper. Those prioritised were the centrality of family, continuity of care and wanting to be treated as unique, yet wanting the same thing (e.g. to be listened to and helped to communicate), despite differences. The importance of financial barriers to care, and barriers to talking therapy were also voiced.

In December 2020, a small group of 3 patient representatives who were part of our larger programme of work discussed our emerging themes, and confirmed that they resonated with them, but directed us to be cautious in interpreting the role of faith (see discussion section for a full account). We know that religion is in decline in England and Wales, with the 2021 census showing a 13 percentage point drop of those identifying as “Christian” to below half of the population [50]. Additionally, the second most common reporting is now of “no religion”, up 12 points to almost 4 in 10 participants. Of Muslim identifying participants (who are most likely to include racialised minorities amongst them), there is an increase by 1.5% to 6.5%, with the highest proportion being in Tower Hamlets in London (about 4 in 10). Additionally, there is variation in religiosity amongst different racially minoritised groups in the UK, with one survey finding almost 2 in 10 without a religion, and uneven religiosity amongst different groups (e.g. relatively high in Pakistani groups) [51].

Finally, in June 2021, themes were similarly presented to a group comprising three patients and a carer (from our existing PPIE contacts as well as advertising in the *Breast Cancer Now* bulletin). Here, participants of Japanese, Pakistani, Bangladeshi and Kenyan heritage confirmed our themes but pointed out, that regardless of barriers like stigma, peer support was important for racially minoritised participants. In the 2^nd^ and 3^rd^ consultations (both online due to the coronavirus pandemic), we presented our findings in considerably more detail, as they were more developed by then. This prompted richer discussions. PPIE directions are touched upon throughout this paper where the PPIE contributors wanted to emphasise or clarify a theme.

### Study characteristics and quality

**Fig 1:**
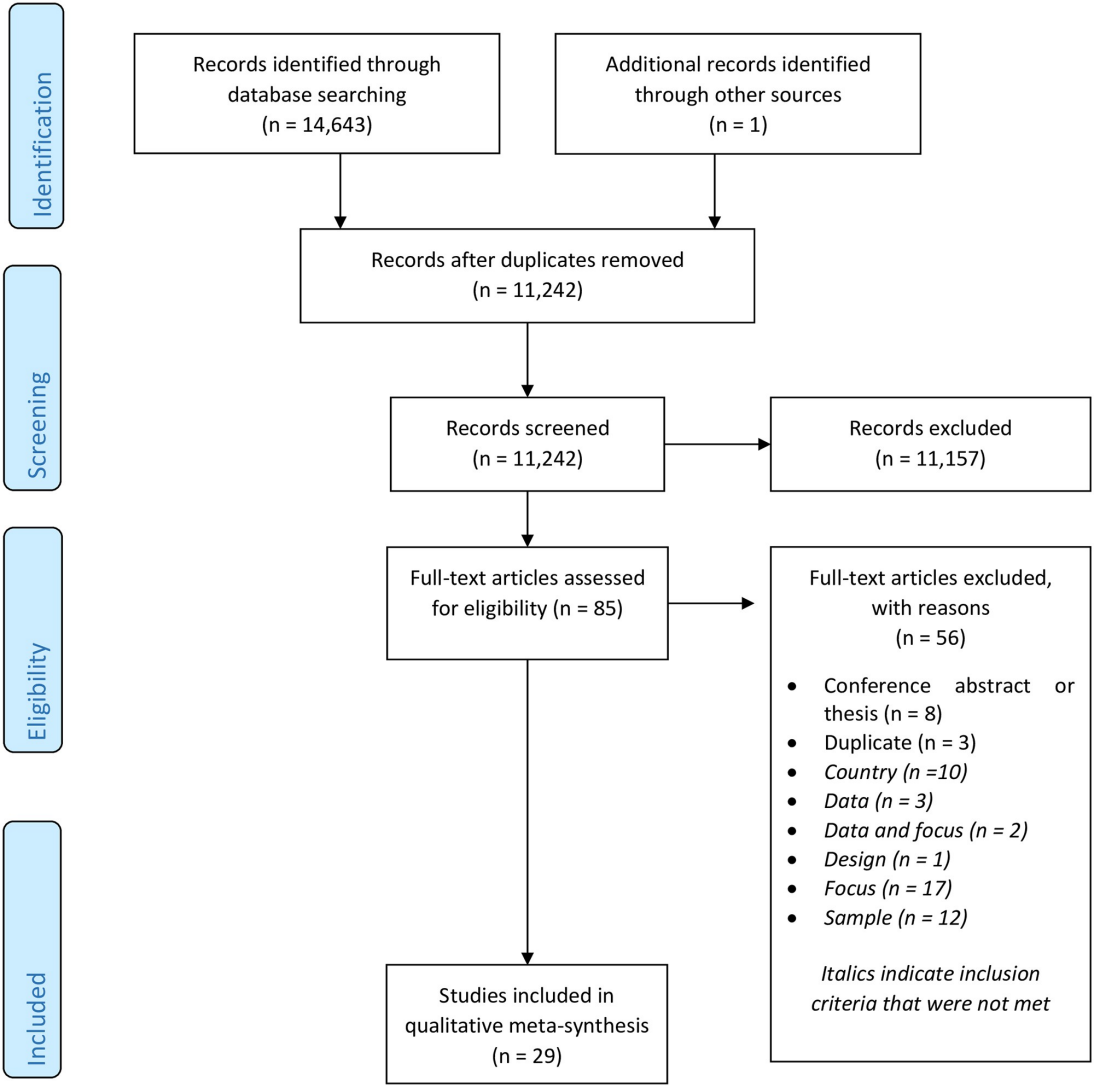
PRISMA flow diagram of study selection for the meta-synthesis.

In order to inform a specific research trial, we focused on studies published between 2010 and 2018 (see limitations section), of which there were 29 relevant papers (see Fig 1 for an outline of the full selection process for studies, including numbers of papers selected at each stage). All included studies focused on cultural and social influences in health care experiences and encounters. Seven focused on cancer (4 on breast cancer, 1 on prostate cancer and 2 on mixed cancers), while the rest covered mental health (7 in people with depression, 4 in schizophrenia and psychosis and the remainder involving people with various or non-specified mental health problems). The studies reflected a range of care settings; 11 (6 mental health and 5 cancer-focused studies) recruited participants via a range of mainly non-NHS community groups/services including charities and support groups, while 9 recruited participants via community mental health services. One cancer study recruited participants via hospitals, while 5 mental health studies utilised a mixture of health care settings for recruitment including hospital, primary care and community-based mental health services. The remaining 3 studies (2 mental health; 1 cancer) recruited participants via NHS and community routes.

The ethnicity of participants varied, with the majority being Black African, Black British, Caribbean or South Asian. One study included Eastern European immigrants. Ethnic groups were self-defined in 9 studies, and country of origin was used to define ethnicity in 13 studies. One study selected participants whose first language was Punjabi or Bangladeshi, while limited details were reported on how ethnicity was assigned in the remaining studies. There was one instance of two papers derived from the same primary study [52, 53], and both papers were included and have been treated as separate studies as the focus of each differed. S1 Table outlines the characteristics of the 29 studies included.

Less than half of the included studies met seven or more of the ten JBI criteria, with only one study meeting all ten. When we removed the quotes, themes and subthemes from the studies that did not meet seven or more of the JBI criteria in this way (which removed all those studies where there were additional issues with quality), our findings did not change. All 29 articles met the criterion relating to the adequacy of the conclusions, which is premised on whether the conclusions can be seen to emerge from the analysis or interpretation. All but one met the criterion that related to ‘believability’, which is about whether participants voices were adequately represented [54] (p. 1737, 1739).

### Key recurring concepts

The studies varied in terms of the participant groups and the health care service or intervention experienced. However, the focus of the studies was either primarily (or had a significant element of focus) on cultural and social aspects of the health care encounter. We found that similar concepts and themes arose from studies in mental health and in cancer in relation to culture and ethnicity. For transparency and to aid interpretation of the findings, we have added (C) to indicate a cancer (rather than mental health) focused study, while unmarked studies relate to mental health. “Participant” indicates that a quote used in the results is reported directly from a participant rather than author. Some subtle differences were apparent between different groups and between those with severe, enduring mental health issues (e.g. psychosis and schizophrenia) and common mental health disorders (e.g. depression). These are addressed where relevant in the more detailed explanations of the main themes in the findings section.

S2 Table shows the overarching themes and recurring concepts, with selected examples of first- and second-order constructs. The examples of first- and second-order constructs were purposively selected for their relevance and diversity. All studies containing each theme are listed in the table.

## Findings

The key cultural and social influences on the uptake of health care interventions are played out in different contexts in that they relate to: intricacies of the patient-practitioner relationship (incorporating language and communication challenges participants face in health care interactions); how participants make sense of their illness; how the family can operate in supportive and/or constraining ways; the integral role spirituality or religion can have in coping with life; different types, sources and levels of support; and the consequences of stigma, for both the individual and their family. Each is now considered in turn.

### 1. Patient-practitioner understandings and relations

The sharing of language and conceptual understandings around cancer and/or mental health conditions cannot be assumed in consultations [55] (C) [56] (C) [33] (C) [57] (C) [58–64]. Participants in studies, for example, may not talk about concepts like ‘depression’ as it is not known about within their social networks. One first generation African-Caribbean man revealed how a diagnosis can come as a revelation as prostate discussions do not naturally emerge from his networks. He said of his cancer, “Most of us didn’t know about [the] prostate, what’s it for you know…” [33] (C, participant, p. 65). It may only be after their diagnosis that other relatives or friends who have also had the condition are recognised in retrospect. Clearly, some concepts used by professionals are not only “difficult to understand” [65], but they clash with worldviews prevalent in lay networks. For example, the Western approach of focusing on individual and internal sources of distress, rather than how social relations and structures cause distress, can make little sense. One participant made an impassioned plea, “These problems are real, and I am not really sure that these emotions should be silenced…*the system creates this sort of fears*” (our emphasis) [58] (male participant from Eastern Europe, p. 14). Similarly, the prospects of working towards any recovery was frequently cast in connected terms, as this participant went on to say: “Therapist is like a part of your family it’s important to have a relationship with the therapist” (p. 15). But relations can also inhibit communication, for example, deference to the authority of doctors can inhibit the asking of questions by first generation African-Caribbean men [33] (C). Here, assessing the level of acculturation to Western ways of thinking can be important, as there is much variation between different age groups, migration generations and different racialised minority groups [66].

When elaborating on the importance of relations, participants frequently spoke passionately about how it was critical for them to ‘be seen’ by professionals. That is, to have their whole selves and circumstances in which they lived respected and cared for by practitioners. This could variously involve a range of issues, like acknowledgment of different religious practices, traditional systems of health, as well as understanding of experiences like immigration [52, 53, 58, 67–69]. One Bangladeshi participant believed their practitioner was so good at connecting, that they shared emotions with them, thus they felt safe to be open, “Even though she is a white person, I know that she sympathises with me…When I am uncomfortable you can tell that she feels my pain…” [67] (participant, p. 12). An asylum seeker from Sudan talked about the affective quality of warm genuineness in relationship with a professional that allowed them to be open about their difficulties, “if she has not won my love, some of the things, it’s not easy to talk about it…she’s concerned with my life.” [61](participant, p. 585). For participants, being able to relate to practitioners in useful ways translates into professionals being non-judgemental and empathic, and being able to grasp the affective, relational and social circumstances in which they lived [53, 61, 63, 66, 67, 70–73]; [57] (C) [74] (C) [35] (C). Lack of ability on the part of professionals to connect with the emotional realities facing participants was linked to unfavourable experiences of health care [75](C) [33] (C) [35] (C).

A general lack of recognition of the everyday struggles of racialised minorities creates barriers to care [53, 63, 69], with one mixed raced participant of Caribbean descent arguing, “White people don’t have any real understanding of the experience of not being white in our society… [I] don’t feel like there is an understanding that I have with somebody who is Black.” [76] (female participant, p. 68). Here, the common reflex in health care to discourage “open conversations about race and culture between people of different cultural backgrounds” is not supported by participant accounts (p. 68). Awareness of practitioners’ “own cultural positioning” in relation to patients [73] (p. 180), is helpful if white professionals are to win the trust of minoritised patients. Thus, discussions on race can help engage diverse patients, yet such discussions need to acknowledge the racism that patients face [66, 76].

Positive alliances can be facilitated by cultural or religious matching of the patient and practitioner, so that insider understandings can be shared, although matching is not always welcomed [73]. Matching (e.g. for Muslim religion) can promote a “closer fit in understandings” [65] (p. 13), but is not always acceptable, for example if taboo subjects like sex need to be discussed [65]. Additionally, a practitioner who is able to connect affectively with patients can cut through the need for matching either culturally or religiously [60, 67]. The ways in which agency among racialised minority participants plays out in patient-professional interactions is important [63, 65, 66, 76, 77]. While some participants can exert their preferences, this was not always a widespread practice [75] (C) [33] (C). Some South Asian participants, for example, appreciated authoritative approaches from their professionals [66]. Interactionally, this can translate into reticence in their health care encounters [63] [33] (C) [35] (C).

### 2. Complex and diverse illness attributions

The studies covered a diverse range of racially minoritised people, the different social circumstances in which they lived, and participant experiences with health care. In our affective relational framework, selves are necessarily emergent from their social relations. Thus, complex, multiple and contradictory views on the causes of illness, whether mental illness or cancer, were expected [75] (C) [60, 69, 72]. Participants hold “multiple explanatory models” for causes of illness [60] (p. 749), including medical, spiritual and social accounts [69] (p. 79), which could be “competing and contrasting as well as interchangeable” [60] (p. 744). If selves are primarily interactants as we argue, with fluctuating, varying and conflicting ways of interpreting illness, not surprisingly “flexibility in accommodating multiple approaches” to health conditions was the expectation that many participants brought with them to professionals [72] (p. 126 & 122). However, participant experiences of health practice tended to be relatively narrow in focus, and professionals appeared to struggle to accommodate alternative readings of illness and causality [52, 53, 78]. Mental illnesses, for example, might be interpreted by participants as having a social explanation as explained above, or be thought of as connected to issues of spirituality [52] (p. 169) [72]. Interestingly, some participants highlighted professional deficits which contributed to them withholding information in relation to practitioners (e.g. "we can’t blame them because they’re upbringing is like westernized, they can’t understand if we talk about Jinns [A spirit world being with powers of influence] …” (British Pakistani male)) [60] (participant, p. 747).

The studies revealed (as confirmed by contributors in one of our PPIE groups) that the hitherto frequently hidden affective relational world, elaborated in our results, that participants inhabit, is a moral one. Thus, illness can be seen as a personal test sent from God. A somewhat fatalistic cosmology was common in papers reviewed, whereby forces beyond one’s control were seen to shape health and illness, e.g. “part of her fate” [74] (C) (p. 12), as the will of God [75] (C); [57] (C); [74] (C); [69]; [73]; and to do with karma [55] (C); [57] (C); [74] (C). Here, punishment for previous wrong-doing had wide currency [75] (C); [55] (C); [78]; [57] (C); [74] (C); [66, 69, 72]. Commonly, particularly in mental health studies, supernatural or spiritual entities populated participant worlds, being non-human interactants that participants had to contend with, establishing hazards and opportunities to navigate. Thus, jadu and korni (sorcery), demonic possession, djin/jinn/jiin (spirits), evil eye, witchcraft, black magic, obeah (a Caribbean kind of sorcery) or waswasah (evil satanic whispers) were variously mentioned as perils to be traversed, e.g. “…we pray to our god to make them [waswasah] go away…”) [62] (Asian British Indian participant born in UK, p. 485); [52, 53, 60, 61, 66, 69, 72, 78].

### 3. Drawing strength from faith, spirituality and religion

How spiritually inclined participants connected to their faith—and their associated faith-based communities—influenced the support they believed they received for their condition. In our affective relational model, faith-based approaches generally involve interacting with non-human others in a bid to transform emotions and circumstances. Such interactions were remarkable among spiritual and/or religious participants for their widely attributed beneficial role, transforming subjectivities: By providing reassurance [72]; “[giving] peace of mind” [69] (Muslim participant, unspecified ethnicity, p. 80); helping with managing or coping with health-related problems [56] (C) [33, 78] (C) [57] (C) [74] (C) [66, 69, 72]; and as promoting contentment, emotional strength or positivity. For example, prayer as a kind of communication with a higher power was connected to real world outcomes, e.g. “I pray a lot and feel calm and feel positive from it” [75] (C) (female, born in Jamaica, p. 198). Some believed that their faith played a role in overcoming cancer, e.g. “God gave me this cancer but along with that he sent me very good treatment and support and gave me strength that I needed to get over it” [74] (C) (female, Indian from Kenya, p. 13) [69]. Relationally, faith could present conflicts, such as when accessing services was viewed as a “betrayal of their religion” [69] (p. 81). Prioritising their relationship with a higher power above others, Muslim participants might, for example, qualify their use of a health service for healing with the phrase ‘*Inshallah’* (i.e. if Allah wills it, the result is with God) [78] (p. 266).

While participants might draw strength from their vertical relationship to a higher power, horizontal links with religious leaders or spiritual healers were also critical [69], especially since their recommendations could be preferred to those of health professionals [53, 66]. Such individuals might be approached either before medical help was sought [60], or “in parallel with medical treatments” [72] (p. 122). Aware that such approaches might be disapproved of in Western medicine, participants attempted to present themselves in a favourable light, including by hiding or alternatively defending their faith to professionals, who seemed to them to lack understanding or information. Some participants, for example, had to explain to professionals that “someone, suddenly going into…speaking in tongues” was not necessarily a mental illness, but rather something that “from time to time…happens” [60] (unidentified participant, p. 748). Religiosity also connected patients via “associated group support” or institutionally, such as via churches or mosques [75] (C) (p. 198). In affective relational terms, faith can be considered a “continuous thread” running through the social lives of many (but certainly not all) participants included in the studies: A kind of non-human “force which sustained them” in their ongoing struggles and “hard lives” [33] (C) (p. 69). Thus faith and faith-based communities offer a range of emotional, practical, social and spiritual supports [59] [56] (C) [33] (C) [57] (C) [72], despite frequently being ‘othered’ in contemporary health care.

### 4. Sources of support

Professional services are not always positioned by participants as the obvious answer to their health-related problems. A ‘hierarchy of help-seeking’ develops in informal relations, whereby the relationship to self is frequently a priority, e.g. “…I can get it [help] but you have to help yourself…” [59] (Black Caribbean woman, mental health, p. 14). Here, accessing services for mental health—as opposed to cancer—raised additional issues amongst some groups [63, 66]. For example, many Black women can labour under community-based stereotype that they should be strong enough to deal with things on their own [59, 63, 66]. Thus, there are implications for health, if for example, women attempt to not “allow themselves to get depressed” [59] (Black Caribbean woman, mental health, p. 13). Or when men attempt to remain positive and in control, e.g. “I am a man, I can sort it out [myself]” [63] (unidentified male in focus group, p. 4). As above, relating to the self is gendered, but there are affective dimensions too, like the emotional effort involved in “using ‘positive thinking’” or accessing “‘inner-strength’”, which could require the cultivation of internal qualities like patience in particular [69] (author discussing diverse Muslim participants, p. 81). The endeavour of avoiding shame was highlighted by Eastern European immigrants to the UK, because “people learnt to be strong”, and turning to services could be considered a sign of weakness [58] (participant, p. 10).

Family or friends are a “first point of call for…participants who failed to resolve difficulties by themselves” [58] (p. 12) [75] (C) [63, 69]. Further, wider social circles could be called upon if required [57] (C) [74] (C). With respect to professionals, one diverse group of Muslim participants considered that mental health services should be reserved “for serious issues” [69] (p. 88). Some Black Caribbean women articulated it as professionals being helpful when self-help and lay networks “failed to meet their needs”, although few women in this sample actually sought such help for their mental health [59] (p. 14). There is also the issue that not all participants endorse medical services for their condition, for a range of deeply felt reasons (e.g. “Social stigma and shame…mistrust of services and lack of understanding…of the symptoms and causes of mental illness”), and so they become particularly reliant on their family and friends [60] (p. 749). Highlighting the importance of affective dramas which play out behind the scenes, lay networks can be seen as superior to dealing with emotional distress compared to health services which can be seen as unsafe. One Black Caribbean female said of a family member in a study of perinatal mental health, “How we managed to get her out of that deep cloud was by giving her a lot of support… if she had gone down the tablets route, she would definitely be institutionalised by now” [70] (participant, p. 259). Highlighting the notion of selves as emergent from relations, family encouragement to access professional services was considered vital by some participants before they sought help [58, 63].

There are complexities to consider when accessing lay support. PPIE contributors of our study emphasised that while families may be able to provide practical support, they might struggle to provide emotional support. Those with an illness might try to protect their families from the condition, while those without local family and friends can become especially isolated [33, 63, 79] (C) [57] (C) [53, 61, 62]. When factoring in the emotive reality of accessing professional services (e.g. fear of stigma), on top of the limits to lay support, one study described how South Asian mothers with postnatal depression could become “extremely isolated and desperate for support” [62] (p. 487).

Complementary and alternative approaches to medicine, such as meditation or massage, some of which could be accessed via mainstream services in the context of cancer, (e.g. “Aromatherapy for an hour is very relaxing”), can be desirable [58] (immigrant from Eastern Europe, p. 10). There are a range of traditional remedies that are passed on via families, and which participants draw on. In one study, African-Caribbean men who had experienced prostate cancer used remedies from “the family armamentarium… bitter tasting bush and herbal teas (black tea, black mint, jack in the bush, fever grass, cerasee) that were prepared by their mothers” [33] (C) (p. 65).

If participants did access health services, they highlighted the pain involved when professionals did not engage with them and their experiences respectfully [62]. In terms of our affective relational model, it is critical for participants to be recognised by professionals in all their humanity, if they are not to find themselves ‘othered’ and disempowered in health care. Participants yearned for practitioners “who will listen to us, who will allow us to talk” [63] (unspecified participant, p. 5) [70]. However, such calls highlighted the inadequacy of mainstream provision, since appropriate talking therapies were either not readily accessible [53], health professionals were unaware of the particular services available, or professionals were yet to “[widen] their cultural knowledge of what was considered normal” in a way that would make useful therapy options available to patients [63] (unspecified male participant, p. 5).

### 5. The role of family

Participants’ accounts of health and care strikingly emerge from within family interactions. Families frequently play complex supporting roles during illnesses, including a facilitating role in terms of accessing services; providing support throughout the illness [33] (C) [57] (C); and more specifically, helping with practical issues (e.g. childcare). Dramas can play out, such as when families attempt to bridge service gaps, e.g. “She [mother] had to fight to get him [brother] assessed and they were telling her ‘no, there is nothing wrong […]’. From the first time he was sectioned after an overdose. They were not there…” [63] (unspecified male participant, p. 5) [74] (C) [72]. As well as facilitating access, families may mediate with community-based services in the case of mental illness [72]. Transactionally, participants report being encouraged, advised or urged to seek help by family members [33, 58] (C) [69]. Here, family views may be considered essential to elicit [66]. However, for some South Asian women, while family discussions could help them with decisions about service participation [67], family members were also portrayed as having the power to grant or withhold permission to attend services e.g., “he [husband] allowed me to go” [71] (Pakistani participant, p. 4).

The emergence of complex decision-making embedded in family relations had profound implications for health care. Participants in included studies described how their networks of family and friends could provide a feeling of a “safe framework” for discussion—and decisions—around health problems, such as mental health crises [63] (p. 3). Sometimes, such was the sense of threat from outside, that the approach taken meant that “everything stays in the family” [58] (p. 8) and problems are dealt with “in-house” and “we make decisions as a family” [69] (Muslim participants, p. 81). There are considerable variations, however. For example, in some matrifocal communities (e.g. Black communities/African-Caribbean/ Black Caribbean), women may feel a need to be strong, and not upset family equilibrium [55] (C) [66, 70], potentially restricting the scope for discussion of their own issues. In some households (e.g. Eastern European, Asian), the discussion of certain illnesses (e.g. cancer, mental illnesses) were actively suppressed, primarily due to the profound stigma and secrecy in their communities [55, 58] (C). As one participant recounted, “I just said I’ve got a lump in my breast. And then my mother said “She’s saying she’s got cancer” and my father replied “No-body mentions that word in the [Asian] community” [55] (C) (Asian participant, p. 137).

### 6. Stigma and its consequences

The highly sensitive nature of stigma played out behind the scenes of health consultations for many racially minoritised groups. There were some differences according to health condition and community. For instance, the ‘taboo’ around cancer itself could differ between communities (e.g. African/African-Caribbean vs Asian communities, being more overt in the latter) [55] (C). While the family and wider community were seen as integral to support (and in many cases were reported as supportive), having cancer was difficult to discuss in some families because of fears around reactions in wider networks that could make whole families vulnerable [57] (C). Cancer stigma can evoke extreme consequences in some communities, like social exclusion (e.g. “And in India…a personal ad [for an arranged marriage will say], nobody with [a disease] need apply”) [55] (C, Asian woman, p. 137). There were fears that news of cancer could spread out into communities. While our PPIE contributors underscored the importance as well as dread involved in accessing peer support, the studies we reviewed tended to emphasise the trepidation “…what happens if you get there [support group for breast cancer] and you know somebody there?” [35] (C, born abroad Black African woman, p. 9).

There was a similarly disturbing backdrop for mental illnesses, where the fear of stigmatising reactions from communities could be profound [60, 66, 68]. Stigma encouraged people to hide their diagnoses from their communities [52, 63], meaning that participants suffered “in secrecy” so as to avoid shame [58] (p. 10). Here, gossip on the “community grapevine” was especially highlighted by South Asian Muslim participants [66] (p. 521). For some communities, the potential for disgrace associated with mental illness not only applied to participants, but could also extend out into immediate families and beyond, e.g. “you lose trust in this person and whole family, maybe relatives as well…” [63] (focus group participant, ethnicity undisclosed, p. 4). Thus, even the whiff of psychological approach in health care (e.g. cognitive behavioural therapy) could be “quite intimidating, quite scary, quite daunting” [65] (participant, second generation Pakistani Muslim man, p. 12). Mental illness in some communities continues to be an off-limits topic: “Stuff like that is never talked about” [63] (participant from Africa, p. 4), contributing to isolation and a lack of language for mental health [77]. Here, accessing mental health services may compound a sense of isolation, and may be considered a sign of weakness [58, 61]. The stigma around mental health was seen to be further complicated by discrimination faced by racially minoritised cultures in a white dominated society [69]. Indeed, some participants suspected that their poor treatment was linked to their minority status, e.g. “[the doctor said] basically you are wasting our time, and they were horrible…I don’t know if they would have said that if I was white” [62] (woman of Pakistani origin, p. 487).

## Discussion

This is the first qualitative meta-synthesis to elaborate on an affective relationality framework conceptually, to better understand the cultural and social influences on experiences of health care interventions amongst racially minoritised participants. Our synthesis revealed a world of emotions, drama, vulnerabilities, and connectivity that played out (largely behind the scenes) in health care interventions. Until now, these dynamics have not been adequately elucidated and conceptualised in the literature on racialised minority experiences. While these emergent affective meanings and dynamics were frequently thought to play out below the radar of professionals, they nevertheless profoundly shaped the potentials—and limits—of health care interventions. We found interactions and their emergent qualities were vast in terms of their scope to shape health care encounters. They not only framed—but also brought to life—the potentials for selves, such as when decision-making emerged from families. Or via the high stakes nature of stigma that created safety as well as defensiveness in families. Or the way participants in the studies related to themselves, which also influenced the possibilities of care, such as when Black men and women attempted to cultivate internal affective subjectivities like positivity and strength respectively. Or the way participants related to a higher power, so as to cultivate affective states like calmness, acceptance and greater peace of mind. Affective meanings that require the participation of others (like love, shame, safety and recognition-or lack thereof) were especially important for participants in their collaborations with health practitioners. Here, institutional approaches that fail to appreciate the centrality of creating safety and affection in care consultations risk inadvertently ‘othering’ racialised minorities. Our PPIE consultations confirmed the need to interpret health care through the lens of affective relations, for example, by humorously invoking notions of a kind of marriage with their own health care professionals when discussing our findings.

We have been encouraged to consider ourselves in hyper-individualistic ways, for example, as entrepreneurs capable of supporting our own wellbeing [80]. However, our ‘animated via affective relationality’ framework suggests that individuals have needs to be fully recognised and supported by others. Thus people become especially vulnerable in seeking help, when their needs become especially acute. Affective dramas are invoked and alleviated by non-human interactants (e.g. via social institutions like the NHS or religious organisations), such as when illness is interpreted as a test sent by God, or when racialised minorities suspect—but cannot prove—racial discrimination in their health care. Our findings resonate with the rise of relational scholarship emerging in a number of health-related fields, from disability studies to suicidality [12, 81]. Our synthesis suggested that as well as selves emerging interactionally, individuals are not clearly bounded, and so it makes sense they might absorb conflicting ideas about the causes of illness, or consider emotions as shared (e.g. “she feels my pain”) and even caused by others (e.g. “the system creates this sort of fears”). This self is helpfully thought of as a dialogical one [82] (p. 90), whereby the link between the self and society is created as a “multiplicity of voiced positions in the landscape of the mind, intertwined as this mind is, with the minds of other people”. Thus, the self is reflective of the varying interactions, networks and institutions in which it emerges.

Challenging approaches to knowledge that might ‘other’ racially minoritised participants (e.g. as having ‘superstitions’), findings in the studies instead identified the limitations as belonging to Westernised practitioners and health care, e.g. “we can’t blame them because [their] upbringing is like westernized…”. Authors of several papers highlighted that many participants were able to create positive affective subjectivities via their relationship with a higher power. Spiritually inclined participants, for instance, said that engaging in prayer could help them deal with the perceived hazards in their worlds animated by dangers like sorcery or malevolent spirits. Prayer thus modulated their affective subjectivity, such as by promoting reassurance. Intervention by a higher power was thought to create the “strength” to cope. The uncomfortable fit of health care with spiritual beliefs was acknowledged by participants, for example, in their reluctance to open-up to professionals about their spirituality for fear of being rejected by another.

The NHS was originally shaped by religious (and thus moral) thinking in some ways (arguably, the Christian values of the Good Samaritan, equality and the collective good influenced the birth of the NHS) [83]. Nevertheless today, religious people are at risk of being othered as difficult or ‘inconvenient’ users of health care [84]. While British psychiatry allows for considerations of spiritual topics in the assessment and care of people, there are limits. The use of spirituality/religion for *therapeutic* purposes is controversial, with particular concerns about boundary violations [85]. However, our synthesis suggests that at a minimum, clinicians need to be aware of the complex (and potentially positive and negative) role of beliefs and spirituality for many of their patients. Clinicians thus should be prepared to at least negotiate complex content to do with patient existential concerns [40].

While some PPI members stated that faith helped them get through their treatments, it was also pointed out that faith may be less important to young people, and that researchers may choose to ask faith-based questions, rather than allow the topic to emerge naturally in interviews. Information about the study topic guide and/or interview questions was reported in two-thirds of papers under this theme. In 6 of the 8 papers that did report such information, aspects of religion/ spirituality were included in the study topic guides/ interview questions. Nevertheless, spirituality and related concepts were a strong theme reported in 13 of the 29 papers. Additionally, 6 of these 13 papers included participants under 30. The 13 studies also included various ethnic groups, as well as participants in cancer and mental health studies. There were a range of aspects included under this theme, including personal beliefs, consulting with faith leaders and healers, and interactions with faith communities. Nevertheless, there is clearly potential for a more nuanced investigation into the varying importance of spirituality among racialised minorities in future health care research.

The studies uncovered the challenges for participants in bringing their whole authentic selves into health consultations, such as when they suspected discrimination at play, including in regards to mental health care [66], and cancer care [56]. Here, anxieties amongst participants about raising issues of racism are matched by fears practitioners have discussing racism with their patients [86]. This is an especially charged issue as the prioritising of white approaches and knowledge is thought to be institutionalised in health care (i.e. systemic, embedded in ways of thinking and doing things, theories, regulations and laws), and thus pernicious [87, 88]. Despite ongoing debates about the exact roles of institutionalised forms of racism in health [72], we know that it can contribute to adverse experiences of care [89]. Here, hidden values and practices systematically undermine the prospects of people of colour [90]. In psychological therapies, for example, sweeping race-related issues ‘under the carpet’ risks patients not feeling seen, and patients becoming affectively disengaged [66]. Or patients feeling they have to somehow compensate for the lack of skills in their professionals (e.g. “humouring the therapist so that they…give me the help that I really need” [63]). Here too, minorities can find themselves positioned as in need of permission from Westernised health care practitioners, who then become gatekeepers of valuable personal rewards [91], like recognition [92].

Finally, the potential for stigma was deeply embedded in networks, and generated a great deal of fear for participants, powerfully shaping behaviours, for both cancer and mental illness care. The consequences of stigma could be considered so profound that behaviours like help-seeking beyond the immediate family for illness were powerfully curtailed for fear of consequences to self and loved ones (e.g. “you lose trust in…[the] whole family”). Our PPIE contributors, in responding to these results, emphasised that professionals underestimate the role and profound impacts of stigma, another example of how emotion remains hidden from view, despite the way it brings to life health care experiences. One recent paper suggests that professionals not only miss the significance of stigma, but may also contribute to stigma narratives themselves, such as by stereotyping minoritised groups [93].

## Conclusions

Our paper has not analysed the vastly different cultures of participants in our studies, nor have we focused on ‘cultural matching’ or ‘cultural competence’ training among practitioners. Instead, we have focused on interactional aspects of care in our analysis. Our findings suggest that practitioners could be fruitfully trained to draw upon their own emotional lives, to improve their connections with minoritised patients. That is, to understand that similar dramas to their own are playing out behind-the-scenes in their care consultations. And to appreciate how such vulnerabilities tend to be hidden for a range of reasons, like lack of safety, shame and fear of rejection. Our findings suggest that elevating the importance of—and respect for—this hidden emotional world of consultations, with legitimisation of racially minoritised experiences therein, could improve care.

In drawing the synthesis together, we took a ‘decolonising attitude’ [4], i.e. listening to seldom heard voices, being inclusive of differences (e.g. cultural, gender) and avoidance of ‘othering’ social groups [94]. A key way to ‘decolonise’ research is to accept that where our “concepts are at odds” (*ibid*, p. 3) with lived experience, then we need better concepts. Arguably, the ‘relational turn’ in a range of disciplines [95], promotes the decolonising agenda, insisting on the humanity of all whose voices remain unheard [94]. Cultural matching can overcome some of the problems caused by differential positioning, but so too can practitioners who have better awareness of how emotional forces are central to understanding care experiences. Partly, this is about practitioners understanding their positioning in relation to systemic racism [73]. But it also means becoming aware of an underlying world of feelings that emerge in consultations. It seems that rather than teaching competencies in relation to a range of cultures, professionals instead could be trained to understand the central importance of considering connections in care.

Relationality focuses our attention on considerations of power differentials between patients and professionals, and how selves and emotional experiences are emergent from social life, emphasising that we are all vulnerable, whether we acknowledge it or not. This perspective helps us to move beyond the focus on individual patient experiences in health care policy, which can prevent us from seeing how affective experiences are *co-created* by interactions that occur in consultations and institutions (like the NHS). Hidden issues like patient disengagement caused by such problems as ‘racial weathering’ (the accumulation of stresses from micro-aggressions and discrimination) [96] experienced in care, and associated patient fears about bringing their authentic selves into the NHS, are best addressed relationally.

### Strengths and limitations

In order to inform a specific trial of a novel psychological intervention for people living with and beyond cancer, we focused on studies up to 2018. There is no clear guidance on how and when to update meta-ethnographies, nor whether indeed they should be updated [97]. As our meta-ethnography was designed to provide a robust conceptual contribution, we argue that the selection of the time period for reporting is less important than the synthesis itself. To test out this hypothesis, we carried out additional searches and reviews. While our original searches aimed to be comprehensive, our subsequent searches were more purposeful in nature (as recommended in the EMERGe guidance) [31]. Here, we aimed to seek out and test “all available concepts until theoretical saturation was achieved” (p. 7). One author (KP) searched for—and two authors (KP and DR) reviewed—relevant journal papers post the original search, through to February 2023, by searching PubMed, PsycINFO and SocINDEX. Frequently used index terms from the initial set of included studies (see S1 Appendix) were used. Eight relevant journal papers were retrieved, and two near-miss papers were excluded. One excluded paper focused on inequalities, and included three out of 11 participants who were non-white [98]. The focus too was on unfairness and difficult experiences (e.g. domestic abuse and drug use) leading up to participants coming into a mental health ward, rather than focusing on NHS services themselves. The second excluded paper focused on decision-making, rather than treatment experiences [99]. However, where it did touch on treatment in passing, it resonated with our themes (e.g. inclinations for spiritual healing, responsibilities towards families). All included papers fitted the relationality framework we developed, including patient appeals for recognition, the importance of respect and trust in consultations [100, 101], while adding some interesting nuances. For example, one paper emphasised that religion was a place where participants could turn to so as to be heard, e.g. “He [God] hears me when I pray [102] (p. 356). Others emphasised how Black African and Black Caribbean participants tend to put on a positive front to the world, while affectively needing to experience dignity in their health care [103, 104]. Finally, one paper confirmed our PPIE reports that peer support groups could provide safe and valued support for racially minoritised people despite their stigmatising risks [105].

The research team itself represented a diverse range of backgrounds, clinically (e.g. medicine, psychiatry, nursing, pharmacy, psychotherapy, oncology) and academically (e.g. sociology, psychology, primary care, public health, global health, complementary medicine). The team encompassed people from a range of ethnic backgrounds. While the majority of the initial data collection and analysis was carried out by SD, EM and KP, subsequent discussions and debates specifically focused on reflecting on the extent to which the iteratively developing manuscript and conceptual framework ‘made sense’ to minoritised team members. The value of using a quality appraisal tool to assess studies included in meta-ethnographies is debatable [106], and there is no consensus on what is quality in qualitative research. It is suggested that interpretive judgements remain important. Thus, we operationalised the JBI in a broad way (high quality (7+) vs. low, according to the ratings of both researchers).

Although we examined beliefs and perspectives of participants in relation to services and interventions, relationships cannot be fully explained by the views of one side. While beyond the scope of this meta-ethnography (as too many papers would have been retrieved to do the analysis justice), future relational research from the perspectives of both patients and health care professionals might uncover additional themes. With the large number of diverse studies included, we could have carried out a number of different sensitivity analyses and/or grouped the studies in various ways for initial analysis. There were no apparent differences in themes that we detected that could be linked to different settings, contexts, and ways of collecting or analysing data. Only 3 studies solely relied on focus groups for data collection, while the remaining studies all incorporated one-to-one interviews. Additionally, none of the papers report on the effect of group processes or contexts. Therefore, it is difficult to draw conclusions regarding the impact of the group context. However, at least one of the three focus group studies contributed to each of the overall themes. Nevertheless, we did focus on identifying broad themes, divergent cases and differences between ethnic groups throughout the results. Differing severity of mental illness was another aspect we considered during the analysis, and subtle differences were recorded in our findings. We also focused on the patients’ perspectives, and we did not extract findings related to professional perspectives.

Due to the large number of studies identified, we restricted the scope to UK studies from 2010 onwards. Restricting papers to the UK ensured that the findings better related to the NHS context, where the main SURECAN trial was conducted [6]. Cancer survival continued to improve across the UK (and other high income countries) between 1995 and 2014, although between country differences have persisted [107]. Mental health services are also evolving rapidly in the UK, and the post-2010 era coincides with an end to national race equality policy, in favour of a broader approach designed to match the range of identities people possess—and the multiple fronts upon which they may face discrimination—with a focus on personalised care [108]. This shift to building mental health care around people seemed to suggest a different era of care at least from 2010 onwards.

One of the overarching aims of our meta-ethnography was to inform a service design for a cancer-related psychological intervention for use in a large UK trial, which recruits from the NHS, and if successful, could be implemented in the NHS. The NHS is shaped by particular features that distinguish it from other health systems (e.g. currently free at the point of access; influenced by over a decade of austerity, restructuring and health care policies; difficulties in patients accessing care in the COVID era; staff shortages; and intense pressures on staff [109, 110]). This system in the UK contrasts with the private system in the USA (the main source of comparable qualitative international studies); characterised by high relative costs; well-trained workforces; private insurance policies and practices; patient payments (which can vary considerably) and marked inequities in coverage for citizens [111]. Nevertheless, in terms of the central concept developed in the paper, specifically how we need to interpret care through the lens of how it is animated by emergent emotions, our paper is likely to have implications for practices “at a broader level, thus allowing it to speak across realms, sites, and contexts” [112]. Thus, we expect our paper will have lessons for health care interventions more generally outside of the UK.

## Supporting information

S1 Table Table outlining study characteristics. (DOCX)

S2 Table Overarching themes and recurring concepts. (DOCX)

S1 Appendix Search strategies used for databases. (DOCX)

S2 Appendix Data items extracted from studies. (DOCX)

S1 Data SURECAN Meta-ethnography data—Link to the 29 included studies. (DOCX)

S1 Checklist PRISMA 2020 checklist. (DOCX)
