# Supplementary material for: A meta-ethnography investigating relational influences on mental health and cancer-related health care interventions for racially minoritised people in the UK
Source: PLoS One. 2023 May 10;18(5):e0284878. doi: 10.1371/journal.pone.0284878 (PMC10171693; doi:10.1371/journal.pone.0284878)
Supplement: S1 Table — (DOCX) [file pone.0284878.s001.docx]

**TABLE 1: Table outlining study characteristics**

| Authors | Location | Setting | Focus | Participants (number; age range; gender) | Ethnicity* or country of origin or nationality | Language spoken; translator used | Sampling approach | Data collection methods | Data analysis methods | Quality (JBI score 7+) |
| --- | --- | --- | --- | --- | --- | --- | --- | --- | --- | --- |
| Bache et al. (2012) | London | Community | Ethnic influences on coping, help-seeking and cancer care experiences | 8  35-81  5F 3M | B | NR  NR | Not specified (likely convenience) | SSI | Framework | No |
| Barlow and Lloyd-Knight (2012) | London | Hospital and community | Culturally specific concerns of BME women confronting breast cancer detection and diagnosis | 33  20s-60s  33F | AB | Punjabi, Gujarati, English  Yes | NR | FG and follow up interviews | Thematic | No |
| Chtereva et al. (2017) | South of England | Community | Experiences of Central and Eastern European immigrants of immigration to UK and of psychological services | 16  19-69  12F 4M | Poland, Bulgaria, Romania, Latvia, Lithuania | NR  Yes | Purposive and snowball | SSI | Inductive thematic | Yes |
| Dos Santos et al. (2012) | Southwest city | Adult MHS | Experiences of African-Caribbean clients and their white therapists during the process of therapy | 6 (3)  25-42  2F 1M | B | NR  NR | NR | SSI (2 rounds) | Thematic and social constructionist discourse | Yes |
| Edge (2011) | Northwest England | Community | Stakeholder perspectives on low levels of consultation for perinatal depression in Black Caribbean women | 42  18-43  42F | B | NR  NR | Purposive | FG | Framework | No |
| Edge and MacKian (2010) | North of England | Community and hospital | Prevalence and psychosocial risks for perinatal depression and help-seeking in Black Caribbean women | 12  18-43  12F | B | NR  NR | Purposive and theoretical | In-depth interviews | Likely thematic | No |
| Fazil et al. (2015) | Birmingham | Community | Experiences of, and needs for, cancer care among members of a range of minority ethnic groups | 50 (35)  NR  23F 12M | ABW | Mirpuri, Punjabi, Pushtu, Urdu, Gujarati, Bengali, Chinese, English  Yes | Purposive | SSI | Thematic | Yes |
| Islam et al. (2015) | Birmingham | Community | Cultural appropriateness, accessibility, and acceptability of the Early Intervention for Psychosis Services in Birmingham | 66 (22)  18 - 35  11F 11M | ABO | NR  Yes | Purposive | FG | Thematic and framework | No |
| Jackson-Blott et al. (2015) | Swindon | Primary care IAPT service | Evaluating a “resilience-focused” CBT -based psycho-educational course for asylum seekers | 8  17-50  4F 4M | Nigeria, Gambia, Iran,  Iraq,  Kenya, Pakistan, Sudan | NR  No | Not specified | SSI | Inductive thematic | No |
| Littlewood and Dein (2013) | London | Community | Distal causality and providence associated with references to Allah (God) (in the context of mental illness) | 44 (25)  27-65  9F 16M | A | Sylheti  Yes | Convenience | SSI | NR | No |
| Lovell et al. (2014) | Northwest England | Primary care and community | Developing and evaluating an acceptable and culturally sensitive psychosocial intervention for older people, and people from ethnic minority communities | 39 (15)  24-60  15F | AB | Urdu, Punjabi, Hindi, Bangla, Sylheti, Somali  Yes | Not specified | SSI | Thematic and framework | No |
| Lwembe et al. (2017) | West London | Community MHS | Evaluating a pilot cross-sector initiative in co-production approaches to deliver a MHS for black and minority ethnic communities | 18 (12)  NR  NR | NR  NR | NR | NR | SSI and FG | Emergent thematic framework | Yes |
| Masood et al. (2015) | Manchester and Lancashire | Primary care and community | Assessing acceptability and overall experience of the Positive Health Programme by British South Asian mothers | 17  20-45  17F | A | Urdu  Yes | Random | In-depth interviews | Thematic | No |
| Memon et al. (2016) | Brighton and Hove | Community | Perceived barriers to accessing MHS among people from Black and minority ethnic backgrounds | 26  18-65+  13F 13M | ABM | NR  NR | Not specified | FG | Thematic | No |
| Nanton and Dale (2011) | Birmingham | Community | Role of ethnicity in determining experiences of prostate cancer in African Caribbean men | 16  50-83  16M | B | NR  NR | Snowball | Interviews | Thematic | No |
| Patel et al. (2014) | London, Birmingham Cardiff, | Community | Black and South Asian women’s experiences of breast cancer diagnosis and treatment in the UK | 22  43-75  22F | ABM | English, Gujarati, Hindi,  Urdu  Yes | Snowball and chain referral | SSI | Inductive thematic | No |
| Patel-Kerai et al. (2015) | London | Community | Experiences of Gujarati-speaking Indian women, who had previously been diagnosed and treated for breast cancer | 5  55-76  5F | A | Gujarati  Yes | Snowball | SSI | IPA | Yes |
| Rabiee and Smith (2013) | Birmingham | Community | Using and providing MHS from the perspectives of black African and black African Caribbean MHS users, carers, and other stakeholders | 97 (25)  NR  14F 11M | B | NR  Yes | Purposive | FG and SSI | Framework | Yes |
| Rabiee and Smith (2014) | Birmingham | Community | Extent to which MHS provision is moving towards a patient-centred approach: experiences of accessing services among black African and African Caribbean service users and carers | 49 (25)  NR  14F 11M | B | NR  Yes | Purposive | FG and SSI | Framework | Yes |
| Rathod et al. (2010) | Hampshire and London | Hospital and community | Producing a culturally sensitive CBT manual for therapists working with psychosis patients from ethnic minority communities | 114 (15)  NR  6F 9M | AB | Urdu, Patois  Yes | Purposive, targeted | SSI and FG | Thematic | Yes |
| Singh (2016) | Unknown | CBT service | Exploring emotionally charged moments in the person's native language during CBT in English, their second language | 7  NR  NR | NR | Punjabi,  Bangladeshi  NR | NR. Selected from referrals | SSI | Framework | No |
| Tarabi et al. (2018) | England | Community | How second-generation Pakistani Muslim men made sense of their experience of CBT | 6  20-45  6M | A | NR  NR | Purposive | SSI | IPA | Yes |
| Tompkins et al. (2016) | North and South East England, inner London | Hospitals | Comparing the experiences and expectations of a sample of White, Black and South Asian women with breast cancer across major cities in England | 66  34-84  66F | ABWO/M | NR  NR | Non-proportional quota | In-depth interviews | Framework | Yes |
| Vincent et al. (2013) | England and Wales | Hospital and primary care | Acceptability and experiences of trauma-focused CBT for asylum seekers with PTSD | 7  19-42  3F 4M | Sudan, Zimbabwe,  Afghanistan, Burundi, Sudan,  Iraq | NR  NR | NR (likely convenience) | SSI | IPA | Yes |
| Wagstaff et al. (2018) | West Midlands | Community-based MHS | Experiences of men with a diagnosis of schizophrenia, self-reported as ‘black’, with a history of disengagement from MHS | 7  31-64  7M | B | NR  NR | Purposive | SSI (2 rounds) | IPA | Yes |
| Weatherhead and Daiches (2010) | Lancaster | Community | Understanding of Muslims of concept of mental health and how mental distress can best be addressed | 14  28-77  7F 7M | Indonesian, Iranian, Turkish-British, British, Pakistani, Egyptian, Sudanese, Malawian | NR  NR | NR (likely convenience) | SSI | Thematic | No |
| Weich et al. (2012) | Birmingham | Community-based MHS | Users’ and carers’ accounts of recent episodes of severe mental illness and care received in a multi-cultural city | 53 (40)  <25->45  18F 22M | ABW | NR  Yes | Typical case | In-depth interviews | Not specified | No |
| Wittkowski et al. (2011) | Greater Manchester | Community | Experience of post-natal depression in South Asian mothers | 10  NR  10F | A | English, Urdu, Gujarati, Bengali, Punjabi, Hindi  Yes | NR | SSI | Constant comparison and grounded theory | Yes |
| Yon et al. (2018) | London | Community-based MHS | Ways in which therapists in a specialist cultural service challenge core cultural beliefs of a family during therapy | 4 (2)  15 and 40s  IF 1M | A | Urdu, English  NR | Purposely selected | SSI | Thematic | No |

KEY: A: Asian/Asian British; B: Black/African/Caribbean/Black British; M: Mixed/Multiple; O: Other ethnic groups; W: White; CBT: Cognitive Behavioural Therapy; FG: focus groups; IAPT: Increasing Access to Psychological Therapy; IPA: Interpretative Phenomenological Analysis; MHS:mental health services; PCS: primary care services; SSI semi-structured interviews; NR: Not recorded
