# Supplementary material for: A meta-ethnography investigating relational influences on mental health and cancer-related health care interventions for racially minoritised people in the UK
Source: PLoS One. 2023 May 10;18(5):e0284878. doi: 10.1371/journal.pone.0284878 (PMC10171693; doi:10.1371/journal.pone.0284878)
Supplement: S2 Table — (DOCX) [file pone.0284878.s002.docx]

**Table 2 - Overarching themes and recurring concepts**

|  |  | Selected examples | |  |
| --- | --- | --- | --- | --- |
| Overarching theme | Recurring concepts | First-order constructs  (patient/participants’ quotes) | Second-order constructs  (authors’ interpretations) | Source papers for theme |
| **Patient-practitioner understandings and relations**  Not being able to speak the same language may result in difficulties in communication. Lack of a shared understanding of concepts can also present barriers.  Dynamics of patient-practitioner relationship influences experience of, and engagement with, healthcare services.  Professionals’ ability and willingness to listen to (and respect) patients’ beliefs are considered paramount. | Language as a barrier to appropriate communication  (for non-native English speakers) | *When I went the second time and took another person the doctor explained to her and she hid it from me* **(Fazil et al., 2015, p99)** | Communicating through family and friends was reported to have contributed to both relationship and communication problems **(Fazil et al., 2015, p98)** | Barlow and Lloyd-Knight, 2012  Chtereva et al., 2017  Edge and MacKian, 2010  Fazil et al., 2015  Islam et al., 2015  Masood et al., 2015  Memon et al., 2016  Nanton and Dale, 2011  Patel-Kerai et al., 2015  Rabiee and Smith, 2013  Rathod et al., 2010  Singh, 2016  Tarabi et al., 2018  Tompkins et al., 2016  Vincent et al., 2013  Weich et al., 2012  Wittkowski et al., 2011  Yon et al., 2018 |
|  | Limited knowledge of medical terms  (the illness itself, symptoms, associated concepts) | *Most of us didn’t know about prostate, what it’s for you know.* **(Nanton and Dale, 2011, p65)** | Although they knew it to be common among old men, it had been little discussed (in the West Indies) and, as they had been young at the time of their departure, their knowledge had been vague. **(Nanton and Dale, 2011, p65)** |  |
|  | Empathy and sensitivity | *There were times that we did not want to talk and we were shy or hesitant; but she was very encouraging. She made us feel that it is ok and we can open up.* **(Masood et al., 2015, p4)** | Most importantly, facilitators’ skills such as the ability to listen and empathise, encouraging and non-judgemental attitude were recognized as important…... **(Masood et al., 2015, p4)** | Bache et al., 2012  Chtereva et al., 2017  Dos Santos and Dallos, 2012  Edge, 2011  Edge and MacKian, 2010  Fazil et al., 2015  Islam et al., 2015  Lovell et al., 2014  Lwembe et al., 2017  Masood et al., 2015  Memon et al., 2016  Nanton and Dale, 2011  Patel et al., 2014  Patel-Kerai et al., 2015  Rabiee and Smith, 2013  Rabiee and Smith, 2014  Rathod et al., 2010  Tarabi et al., 2018  Tompkins et al., 2016  Vincent et al., 2013  Wagstaff et al., 2018  Weatherhead and Daiches, 2010  Weich et al., 2012  Wittkowski et al., 2011  Yon et al., 2018 |
|  | Cultural and religious sensitivity | *I suppose that it is always more stressful talking about race with people who aren’t of the same race as you. White people don’t have any real understanding of the experience of not being white in our society.* **(Dos Santos and Dallos, 2012, p68)** | Clients ... seemed to have internalised a social understanding of therapy as being exclusively for exploration of internal, psychological issues, rather than external or social ones.**(Dos Santos and Dallos, 2012, p68)** |  |
| **Complex and diverse**  **illness attributions**  People have multiple ways of making sense of why they become ill. | Multiple explanatory models of illness | *My mother believes that cure will come from God, that God will come through and I believe that as well, obviously medicine is here for a reason as well so …* **(Islam et al., 2015, p745)** | Cultural beliefs often competed and contrasted with medical explanations. **(Islam et al., 2015, p745)** | Bache et al., 2012  Barlow and Lloyd-Knight, 2012  Islam et al., 2015  Littlewood and Dein, 2013  Patel et al., 2014  Patel-Kerai et al., 2015  Rabiee and Smith, 2013  Rabiee and Smith, 2014  Rathod et al., 2010  Vincent et al., 2013  Weatherhead and Daiches, 2010  Weich et al., 2012  Wittkowski et al., 2011  Yon et al., 2018 |
|  | Mental illness as a social issue | *I mean there’s lots of things that can make people go off their head; if they haven’t got proper accommodation, if their house is leaking or if their partner’s gone off …* **(Rabiee and Smith, 2013, p169)** | For service users and carers, mental health was seen as a social issue that was not always understood by professionals. **(Rabiee and Smith, 2013, p169)** |  |
|  | Illness predetermined by forces beyond one’s control | *I’ve never questioned why me you know, it’s God’s wish.* **(Patel et al., 2014, p142).** | … some women believed that their cancer was predetermined by forces beyond their control, such as God. **(Patel et al., 2014, p142)***.* |  |
| **Drawing strength from faith, spirituality and religion**  Faith/spirituality/religion frequently forms an integral part of people’s day-to-day lives. It is an important source of support when dealing with an illness. | Faith as a source of emotional support | *Even though God gave me this cancer…he sent me very good treatment and support and gave me strength that I needed…*  **(Patel-Kerai et al., 2015, p13)** | Participants described how faith in God was an important source of support and engaging in religious activities, such as praying, helped them. **(Patel-Kerai et al., 2015, p13)** | Bache et al., 2012  Edge and MacKian, 2010  Fazil et al., 2015  Islam et al., 2015  Littlewood and Dein, 2013  Nanton and Dale, 2011  Patel et al., 2014  Patel-Kerai et al., 2015  Rabiee and Smith, 2014  Rathod et al., 2010  Weatherhead and Daiches, 2010  Weich et al., 2012 |
|  | Faith-based help-seeking  (consulting with religious leaders, faith/spiritual healers) | *Just that … we can’t blame them because they’re upbringing is like westernized, they can’t understand if we talk about Jinns ...* **(Islam et al., 2015, p747)** | They believed that professionals (i.e., care coordinators and doctors) would not understand their views/ perceptions. **(Islam et al., 2015, P747)** |  |
|  | Faith communities as a source of social and spiritual support | *I draw strength from it because I sing in the choir and you know I pray a lot and feel calm and feel positive from it.* **(Bache et al., 2012, p198)** | It was suggested that religious beliefs promote a positive outlook. Some maintained that religion aids acceptance of their destiny. **(Bache et al., 2012, p198)** |  |
| **Sources of support**  From whom (and when) one seeks support when dealing with an illness or distress | Lay hierarchy of help-seeking  (on a continuum from the individualistic to the community-oriented) | *It’s about where you get the strength from to cope, within your structure before you actually go to the GP. It’s in the home… It’s from your mates and your family circle...* **(Edge and MacKian, 2010, p100)** | In line with their social model of depression, women in this study used a lay hierarchy of help-seeking **(Edge and MacKian, 2010, p100)** | Bache et al., 2012  Chtereva et al., 2017  Edge, 2011  Edge and MacKian, 2010  Islam et al., 2015  Jackson-Blott et al., 2015  Memon et al., 2016  Nanton and Dale, 2011  Patel et al., 2014  Patel-Kerai et al., 2015  Rabiee and Smith, 2014  Rathod et al., 2010  Vincent et al., 2013  Weatherhead and Daiches, 2010  Wittkowski et al., 2011 |
|  | Social isolation  (experienced by those with little or no social support networks) | *… I have no-body, my family are in India. I can’t speak English properly, and I can’t read English to fill out forms.* **(Wittkowski et al., 2011, p486)** | These mothers felt extremely isolated and desperate for support…  **(Wittkowski et al., 2011, p487)** |  |
|  | Different help-seeking pathways  (help-seeking via sources that lie outwith mainstream service provision) | [Many participants wanted] *to access people who will listen to us, who will allow us to talk.* **(Memon et al., 2016, p5)** | Many participants […] raised concerns that talking therapies were not readily provided. **(Memon et al., 2016, p5)** |  |
| **The role of family**  Family and friends can be a source of support and can play an important role in uptake and use of health services. | Provide support  (general and practical) | *And my husband would…drop [son] to school, then come to see me and then go see the other son. So my husband was very supportive.* **(Patel-Kerai et al., 2015, p13)** | Family members, particularly immediate family, were described as providing practical support **(Patel-Kerai et al., 2015, p12)** | Barlow and Lloyd-Knight, 2012  Chtereva et al., 2017  Edge and MacKian, 2010  Lovell et al., 2014  Masood et al., 2015  Memon et al., 2016  Nanton and Dale, 2011  Patel et al., 2014  Patel-Kerai et al., 2015  Rathod et al., 2010  Weatherhead and Daiches, 2010  Weich et al., 2012 |
|  | Influential in accessing care  (enabling or delaying access via their support or disapproval) | *I’ve spoken to my husband about it. He said if that is what your doctor has recommended then go and try it.* (**Lovell et al., 2014, p10**) | Particularly in the case of South Asian women, discussion with family members [was important]. (**Lovell et al., 2014, p10**) |  |
|  | Family and friends as a supportive social network  (for discussion and decisions  about health problems) | *…we make decisions as a family…*  (**Weatherhead and Daiches, 2010, p81**) | The importance of family and friends was repeated throughout the interviews as a way of managing problems. **(Weatherhead and Daiches, 2010, p81**) |  |
| **Stigma and its consequences**  The presence of stigma towards mental illness and cancer.  There are profound multilevel effects that stigma may have in the context of healthcare (individual, social standing of family) | Hide / keep quiet about an illness  (as it brings shame) | *And then my mother said ‘She’s saying she’s got cancer’ and my father replied ‘No-body mentions that word in the [Asian] community’.* **(Barlow and Lloyd-Knight, 2012, p137)** | Women found their families were very supportive, but fearful of having “cancer in the family”. **(Barlow and Lloyd-Knight, 2012, p137)** | Barlow and Lloyd-Knight, 2012  Chtereva et al., 2017  Islam et al., 2015  Lwembe et al., 2017  Memon et al., 2016  Patel et al., 2014  Rabiee and Smith, 2013  Rathod et al., 2010  Tarabi et al., 2018  Tompkins et al., 2016  Vincent et al., 2013  Wagstaff et al., 2018  Weatherhead and Daiches, 2010  Wittkowski et al., 2011 |
|  | Consequences of stigma extend beyond the individual | *. And, […] they whisper about the whole family…and they lose respect.* **(Memon et al., 2016, p4)** | …mental health...could stigmatise the whole family, affecting employment prospects and standing in the community. **(Memon et al., 2016, p4)** |  |
|  | Impact of stigma on help-seeking | *I’m too scared to do it [go to a support group].…what happens if you get there and you know somebody there?* **(Tompkins et al., 2016, p9)** | They wanted to forget about cancer and also worried that in attending support groups people would find out they had cancer **(Tompkins et al., 2016, p9)** |  |
