# Supplementary material for: A meta-ethnography investigating relational influences on mental health and cancer-related health care interventions for racially minoritised people in the UK
Source: PLoS One. 2023 May 10;18(5):e0284878. doi: 10.1371/journal.pone.0284878 (PMC10171693; doi:10.1371/journal.pone.0284878)
Supplement: S1 Appendix — (DOCX) [file pone.0284878.s003.docx]

# Appendix 1 – Search strategies used for databases

Ovid MEDLINE search strategy

Ovid MEDLINE (and basic strategy for other databases; those that required significant adaptation are presented below)

1. Qualitative Research/ or interview/ or (theme$ or thematic).mp. or qualitative.af. or nursing methodology research/ or questionnaire$.mp. or ethnological research.mp. or ethnograph$.mp. or ethnonursing.af. or phenomenol$.af. or (grounded adj (theor$ or study or studies or research or analys?s)).af. or (life stor$ or women$ stor$).mp. or (emic or etic or hermeneutic$ or heuristic$ or semiotic$).af. or ((data adj1 saturat$) or participant observ$).tw. or (social construct$ or postmodern$ or post modern$ or poststructural$ or post structural$ or feminis$ or interpret$).mp. or (action research or cooperative inquir$ or co operative inquir$).mp. or (humanistic or existential or experiential or paradigm$).mp. or (field adj (study or studies or research)).tw. or human science.tw. or biographical method.tw. or theoretical sampl$.af. or ((purpos$ adj4 sampl$) or (focus adj group$)).af. or (account or accounts or unstructured or open ended or text$ or narrative$).mp. or (life world or conversation analys?s or personal experience$ or theoretical saturation).mp. or ((lived or life) adj experience$).mp. or cluster sampl$.mp. or observational method$.af. or content analysis.af. or (constant adj (comparative or comparison)).af. or ((discourse$ or discurs$) adj3 analys?s).tw. or narrative analys?s.af. or heidegger$.tw. or colaizzi$.tw. or spiegelberg$.tw. or van manen$.tw. or van kaam$.tw. or merleau ponty.tw. or husserl$.tw. or foucault$.tw. or (corbin$ adj2 strauss$).tw. or glaser$.tw. or semi-structured interview*.mp.
2. (underclass or marginalised or working class or deprived).mp. or Vulnerable Populations/ or underserved.mp. or disadvantaged.mp. or low income.mp. or deprivation.mp. or minority groups/ or minority health/ or asylum seeker$.ab,ti. or (migrant$ or immigrant$).ab,ti. or gypsy.mp. or gypsies.mp. or traveller$.mp. or polish.mp. or ethnic groups.mp. or ethnicity.mp. or ethnology.mp. or Ethnology/ or Ethnic Groups/ or Culturally Competent Care/ or Cultural Competency/ or minority ethnic.mp. or ethnic minorit$.mp. or racial.mp. or (Cultur$ adj (competenc$ or diverst$ or appropriat$ or safety or respect or responsiveness or sensitiv$ or understanding or knowledge or expertise or skill$)).mp. or Transcultural.mp. or Multicultural.mp. or Cross-cultur$.mp. or (BME or black ethnic minorit$ or black minorit$ ethnic$).mp. or (south asian$ or bangladeshi$ or pakistani$ or indian$ or sri lankan$).mp. or (asian$ or east asian$ or chinese or taiwanese or vietnamese or korean$ or japanese).mp. or (afro-caribbean$ or african-caribbean$ or caribbean or african$ or black$ or afro$).mp. or African Continental Ancestry Group/ or (islam$ or hindu$ or Sikh$ or buddhis$ or muslim$ or moslem$ or jew$ or arab$).ab,ti.
3. Cancer.mp. or Neoplasms/ or psychological therapy.mp. or psychological intervention*.mp. or psychological treatment*.mp. or Psychotherapy/ or counselling.mp. or counselling.mp. or mental health services/ or depressive disorder/ or anxiety disorders/ or mental disorders/ or behavior therapy/ or cognitive therapy/
4. (uk or united kingdom or great britain or wales or scotland or england or Ireland or welsh or Scottish or English or irish or New Zealand* or Malta or Jersey or Guernsey or channel islands or Isle of Man or Canada or Canadian or Australia*).ab,ti. or uk.af
5. 1 and 2 and 3 and 4

EBSCO CINAHL

1. MH Interview+ or MH audiorecording or MH interviews or MH Grounded theory or MH Qualitative Studies or MH Research, Nursing or MH Focus Groups or MH Discourse Analysis or MH Content Analysis or MH Ethnographic Research or MH Ethnological Research or MH Ethnonursing Research or MH Constant Comparative Method or MH Qualitative Validity+ or MH Purposive Sample or MH Observational Methods+ or MH Field Studies or MH theoretical sample or MH Phenomenology or MH Phenomenological Research or MH Life Experiences+ or MH Cluster Sample+ or ethnonursing or ethnograph* or phenomenol* or grounded W0 theor* or "grounded study" or "grounded studies" or "grounded research" or grounded W0 analys?s or life W0 stor* or women's W0 stor* or emic or etic or hermeneutic* or heuristic* or semiotic* or data N0 saturat* or participant W0 observ* or social W0 construct* OR post-modern* OR post-structural* OR poststructural* OR postmodern* OR feminis* or "action research" OR cooperative W0 inquir* OR co-operative W0 inquir* or humanistic OR existential OR experiental OR paradigm* or field W0 stud* or "field research" or "human science" or "biographical method" or theoretical W0 sampl* or purpos* N3 sampl* or focus W0 group* or account OR accounts OR unstructured OR open-ended OR text* OR narrative* or life-world OR conversation W0 analys?s OR personal W0 experience* OR theoretical W0 saturation or lived W0 experience* or life W0 experience* or cluster W0 sampl* or theme* OR thematic or observational W0 method* or "content analysis" or discourse* N2 analys?s or discurs* N2 analys?s or "constant comparative" or "constant comparison" or "narrative analysis" or Heidegger* or Colaizzi* or Spiegelberg* or van W0 manen* or van W0 kaam* or merleau W0 ponty* or husserl* or Foucault* or corbin* N1 strauss* or glaser* or semi?structured W0 interview*
2. underclass or marginalised or working W0 class or deprived or underserved or disadvantaged or low W0 income or deprivation or MH Minority Groups or asylum W0 seeker* or migrant* or immigrant* or gypsy or gypsies or traveller* or polish or ethnic W0 groups or ethnicity or ethnology or MH Culture+ or MH Ethnic Groups+ or MH Cultural Sensitivity or MH Cultural Bias or minority W0 ethnic or ethnic W0 minorit or racial or (Cultur* W0 (competenc* or diversit* or appropriate* or safety or respect or responsiveness or sensitiv* or understanding or knowledge or expertise or skill* or adapta*)) or Transcultural or Multicultural or Cross-cultur* or (BME or black and W0 ethnic W0 minorit* or black and W0 minorit* ethnic*) or (south Asian* or Bangladeshi* or Pakistani* or indian* or sri W0 lankan*) or (Asian* or east W0 asian* or chinese or taiwanese or vietnamese or Korean* or japanese) or (afro-caribbean* or african-caribbean* or caribbean or African* or black* or afro*) or (islam* or hindu* or Sikh* or buddhis* or muslim* or moslem* or jew* or arab*)
3. Cancer or MH Neoplasms or psychological W0 therapy or psychological W0 intervention or psychological W0 treatment* or MH Psychotherapy+ or counselling or counselling or MH mental health services or MH Mental Disorders or depression or MH Behavior Therapy or MH Cognitive Therapy or MH Depression or MH Anxiety disorders or MH Anxiety or MH Counseling
4. (TI ( (uk or united W0 kingdom or great W0 britain or wales or scotland or england or Ireland or welsh or Scottish or Irish or New W0 Zealand or Malta or Jersey or Guernsey or channel W0 islands or Isle W0 of W0 Man or Canada or Canadian or Australia*) ) OR AB ( (uk or united W0 kingdom or great W0 britain or wales or scotland or england or Ireland or welsh or Scottish or Irish or New W0 Zealand or Malta or Jersey or Guernsey or channel W0 islands or Isle W0 of W0 Man or Canada or Canadian or Australia*) ) OR AF ( (uk or united W0 kingdom or great W0 britain or wales or scotland or england or Ireland or welsh or Scottish or Irish or New W0 Zealand or Malta or Jersey or Guernsey or channel W0 islands or Isle W0 of W0 Man or Canada or Canadian or Australia*)
5. 1 and 2 and 3 and 4

EBSCO PsycINFO

1. DE "Interviewing" or DE "Interviews" or DE "Grounded Theory" or DE "Qualitative Research" or DE "Discourse Analysis" or DE "Content Analysis" or DE "Observation Methods" or DE "Phenomenology" or DE "Life Experiences" OR DE "Life Changes" or ethnonursing or ethnograph* or phenomenol* or grounded W0 theor* or "grounded study" or "grounded studies" or "grounded research" or grounded W0 analys?s or life W0 stor* or women's W0 stor* or emic or etic or hermeneutic* or heuristic* or semiotic* or data W0 saturat* or participant W0 observ* or social W0 construct* OR post-modern OR post-structural* OR poststructural* OR postmodern* OR feminis* or interpretive or "action research" OR cooperative W0 inquir* OR co-operative inquir* or humanistic OR existential OR experiental OR paradigm* or field W0 stud* or "field research" or "human science" or "biographical method" or theoretical W0 sampl* or purpos* N3 sampl* or focus W0 group* or account OR accounts OR unstructured OR open-ended OR text* OR narrative* or life-world OR conversation W0 analys?s OR personal W0 experience* OR theoretical W0 saturation or lived W0 experience* or life W0 experience* or cluster W0 sampl* or theme* OR thematic or observational W0 method* or questionnaire* or "content analysis" or discourse* N2 analys?s or discurs* N2 analys?s or "constant comparative" or "constant comparison" or narrative analysis or Heidegger* or Colaizzi* or Spiegelberg* or van W0 manen* or van W0 kaam* or merleau W0 ponty* or husserl* or Foucault* or corbin* N1 strauss* or glaser* or semi?structured W0 interview*
2. underclass or marginalised or working W0 class or deprived or underserved or disadvantaged or low W0 income or deprivation or MH minority groups or asylum W0 seeker* or migrant* or immigrant* or gypsy or gypsies or traveller* or polish or ethnic W0 groups or ethnicity or ethnology or MH Ethnology or MH Racial and Ethnic Groups or MH Cultural Sensitivity or MH Ethnic Identity or MH Cross Cultural Differences or minority W0 ethnic or ethnic W0 minorit or racial or (Cultur* W0 (competenc* or diversit* or appropriate* or safety or respect or responsiveness or sensitiv* or understanding or knowledge or expertise or skill* or adapta*)) or Transcultural or Multicultural or Cross-cultur* or (BME or black and W0 ethnic W0 minorit* or black and W0 minorit* ethnic*) or (south Asian* or Bangladeshi* or Pakistani* or indian* or sri W0 lankan*) or (Asian* or east W0 asian* or chinese or taiwanese or vietnamese or Korean* or japanese) or (afro-caribbean* or african-caribbean* or caribbean or African* or black* or afro*) or MH African Cultural Groups or (islam* or hindu* or Sikh* or buddhis* or muslim* or moslem* or jew* or arab*)
3. Cancer or MH Neoplasms or psychological W0 therapy or psychological W0 intervention or psychological W0 treatment* or MH Psychotherapy or counselling or counselling or MH mental health services or MH Mental Disorders or depression or MH Behaviour Therapy or MH Cognitive Therapy or MH Depression or MH Anxiety disorders or MH Anxiety or MH Counseling
4. (TI ( (uk or united W0 kingdom or great W0 britain or wales or scotland or england or Ireland or welsh or Scottish or Irish or New W0 Zealand or Malta or Jersey or Guernsey or channel W0 islands or Isle W0 of W0 Man or Canada or Canadian or Australia*) ) OR AB ( (uk or united W0 kingdom or great W0 britain or wales or scotland or england or Ireland or welsh or Scottish or Irish or New W0 Zealand or Malta or Jersey or Guernsey or channel W0 islands or Isle W0 of W0 Man or Canada or Canadian or Australia*) ) OR AF ( (uk or united W0 kingdom or great W0 britain or wales or scotland or england or Ireland or welsh or Scottish or Irish or New W0 Zealand or Malta or Jersey or Guernsey or channel W0 islands or Isle W0 of W0 Man or Canada or Canadian or Australia*)
5. 1 and 2 and 3 and 4

EBSCO SOCINDEX

1. DE "Interviewing" or DE "Qualitative Research" or DE "Discourse Analysis" or DE "Content Analysis" or DE "Participant Observation” or DE "Phenomenology" or DE "Life change events” or ethnonursing or ethnograph* or phenomenol* or grounded W0 theor* or "grounded study" or "grounded studies" or "grounded research" or grounded W0 analys?s or life W0 stor* or women's W0 stor* or emic or etic or hermeneutic* or heuristic* or semiotic* or data W0 saturat* or participant W0 observ* or social W0 construct* OR post-modern OR post-structural* OR poststructural* OR postmodern* OR feminis* or interpretive or "action research" OR cooperative W0 inquir* OR co-operative inquir* or humanistic OR existential OR experiental OR paradigm* or field W0 stud* or "field research" or "human science" or "biographical method" or theoretical W0 sampl* or purpos* N3 sampl* or focus W0 group* or account OR accounts OR unstructured OR open-ended OR text* OR narrative* or life-world OR conversation W0 analys?s OR personal W0 experience* OR theoretical W0 saturation or lived W0 experience* or life W0 experience* or cluster W0 sampl* or theme* OR thematic or observational W0 method* or questionnaire* or "content analysis" or discourse* N2 analys?s or discurs* N2 analys?s or "constant comparative" or "constant comparison" or narrative analysis or Heidegger* or Colaizzi* or Spiegelberg* or van W0 manen* or van W0 kaam* or merleau W0 ponty* or husserl* or Foucault* or corbin* N1 strauss* or glaser* or semi?structured W0 interview*
2. (underclass or marginalised or working W0 class or deprived or underserved or disadvantaged or low W0 income or deprivation or DE “MINORITIES” or asylum W0 seeker* or migrant* or immigrant* or gypsy or gypsies or traveller* or polish or ethnic W0 groups or ethnicity or ethnology or DE “Ethnology” or DE “RACIAL CLASSIFICATION” or DE “CULTURAL AWARENESS” or DE “ETHNICITY” or DE “CROSS-cultural differences” or minority W0 ethnic or ethnic W0 minorit or racial or (Cultur* W0 (competenc* or diversit* or appropriate* or safety or respect or responsiveness or sensitiv* or understanding or knowledge or expertise or skill* or adapta*)) or Transcultural or Multicultural or Cross-cultur* or (BME or black and W0 ethnic W0 minorit* or black and W0 minorit* ethnic*) or (south Asian* or Bangladeshi* or Pakistani* or indian* or sri W0 lankan*) or (Asian* or east W0 asian* or chinese or taiwanese or vietnamese or Korean* or japanese) or (afro-caribbean* or african-caribbean* or caribbean or African* or black* or afro*) or DE “AFRICANS” or (islam* or hindu* or Sikh* or buddhis* or muslim* or moslem* or jew* or arab*))
3. Cancer or DE “CANCER” or psychological W0 therapy or psychological W0 intervention or psychological W0 treatment* or DE “Psychotherapy” or counselling or counselling or DE “mental health services” or DE “MENTAL ILLNESS” or depression or DE “Behavior Therapy” or DE “Cognitive Therapy” or DE “MENTAL DEPRESSION” or DE “Anxiety disorders” or DE “Anxiety” or DE “Counseling”
4. (TI ( (uk or united W0 kingdom or great W0 britain or wales or scotland or england or Ireland or welsh or Scottish or Irish or New W0 Zealand or Malta or Jersey or Guernsey or channel W0 islands or Isle W0 of W0 Man or Canada or Canadian or Australia*) ) OR AB ( (uk or united W0 kingdom or great W0 britain or wales or scotland or england or Ireland or welsh or Scottish or Irish or New W0 Zealand or Malta or Jersey or Guernsey or channel W0 islands or Isle W0 of W0 Man or Canada or Canadian or Australia*) ) OR AF ( (uk or united W0 kingdom or great W0 britain or wales or scotland or england or Ireland or welsh or Scottish or Irish or New W0 Zealand or Malta or Jersey or Guernsey or channel W0 islands or Isle W0 of W0 Man or Canada or Canadian or Australia*)
5. 1 and 2 and 3 and 4

Web of Knowledge search strategy

Indexes=SCI-EXPANDED, SSCI, A&HCI, CPCI-S, CPCI-SSH, ESCI Timespan=All years

1. TOPIC: (ethnic* psycholog* qualitative)

2. TOPIC: (cultural psycholog* qualitative)

3. TOPIC: (ethnic* cancer qualitative)

4. TOPIC: (cultural cancer qualitative)

1 or 2 or 3 or 4

Refined by: TOPIC: (uk)
