# Supplementary material for: A meta-ethnography investigating relational influences on mental health and cancer-related health care interventions for racially minoritised people in the UK
Source: PLoS One. 2023 May 10;18(5):e0284878. doi: 10.1371/journal.pone.0284878 (PMC10171693; doi:10.1371/journal.pone.0284878)
Supplement: S2 Appendix — (DOCX) [file pone.0284878.s004.docx]

# Appendix 2: Data items extracted from studies

Location Setting Stated aims

Research question Study design Theoretical approach Ethics mentioned

Participant inclusion criteria Participant exclusion criteria Number of participants

Age range Gender

Ethnicity (ONS census groups) Participants' place of birth

Language spoken (i.e. first language)

Disadvantaged / underserved / under-represented group Psychological intervention/ mental health services

Was the intervention/service culturally adapted? Language in which the intervention/service was delivered Translator involved in the study?

Cancer services / cancer-related health encounters Cancer type

Time since cancer diagnosis Cancer treatment status

Self-management / self-help support intervention for cancer

Coping / patient experience, with discussion of health services / interventions Sampling approach

Data collection methods Data analysis methods
