# Supplementary material for: A meta-ethnography investigating relational influences on mental health and cancer-related health care interventions for racially minoritised people in the UK
Source: PLoS One. 2023 May 10;18(5):e0284878. doi: 10.1371/journal.pone.0284878 (PMC10171693; doi:10.1371/journal.pone.0284878)
Supplement: S1 Data — (DOCX) [file pone.0284878.s005.docx]

**SURECAN Meta-ethnography data – link to the 29 included studies**

<https://doi.org/10.1080/13557858.2011.635785>

<https://doi.org/10.1108/EIHSC-10-2012-0013>

<https://uwe-repository.worktribe.com/output/882027>

<https://doi.org/10.1080/14780887.2012.630827>

<https://doi.org/10.3399/bjgp11X567063>

<http://dx.doi.org/doi:10.1080/13557850903418836>

<https://diversityhealthcare.imedpub.com/ethnicity-gender-and-mental-health.pdf>

<https://core.ac.uk/download/pdf/267286582.pdf>

<https://doi.org/10.1177/0022022115575737>

<https://doi.org/10.1108/MHSI-03-2015-0012>

<https://doi.org/10.1080/13648470.2013.853598>

<https://doi.org/10.1186/s12888-014-0217-8>

<https://doi.org/10.1017/S1463423616000141>

<https://doi.org/10.1186/s12905-015-0263-5>

<http://dx.doi.org/10.1136/bmjopen-2016-012337>

<https://doi.org/10.1111/j.1365-2354.2009.01155.x>

<https://diversityhealthcare.imedpub.com/black-and-south-asian-womens-experiences-of-breast-cancer-a-qualitative-study.pdf>

<https://doi.org/10.21767/2049-5471.100024>

<http://dx.doi.org/10.1080/14623730.2013.824163>

<https://bcuassets.blob.core.windows.net/docs/understanding-mental-health-fatemeh-rabiee-khan-130597441035271345.pdf>

<https://doi.org/10.1017/S1352465810000378>

<https://doi.org/10.7748/mhp.2016.e1046>

<https://doi.org/10.1080/09515070.2018.1471587>

<https://doi.org/10.1186/s12913-016-1625-x>

<https://doi.org/10.1017/S1352465812000550>

<https://doi.org/10.1111/inm.12305>

<https://doi.org/10.1348/147608309X467807>

<https://doi.org/10.1007/s00127-010-0314-z>

https://doi.org/10.1080/02646838.2011.639014

<https://doi.org/10.1111/1467-6427.12158>
